# Insilico Functional Analysis of Genome-Wide Dataset From 17,000 Individuals Identifies Candidate Malaria Resistance Genes Enriched in Malaria Pathogenic Pathways

**DOI:** 10.3389/fgene.2021.676960

**Published:** 2021-11-18

**Authors:** Delesa Damena, Francis E. Agamah, Peter O. Kimathi, Ntumba E. Kabongo, Hundaol Girma, Wonderful T. Choga, Lemu Golassa, Emile R. Chimusa

**Affiliations:** ^1^ Division of Human Genetics, Department of Pathology, University of Cape Town, Cape Town, South Africa; ^2^ Aklilu Lema Institute of Pathobiology, Addis Ababa University, Addis Ababa, Ethiopia; ^3^ Institute of Infectious Disease and Molecular Medicine, University of Cape Town, Cape Town, South Africa

**Keywords:** functional analysis, genome-wide association study, severe malaria, genes, pathways

## Abstract

Recent genome-wide association studies (GWASs) of severe malaria have identified several association variants. However, much about the underlying biological functions are yet to be discovered. Here, we systematically predicted plausible candidate genes and pathways from functional analysis of severe malaria resistance GWAS summary statistics (*N* = 17,000) meta-analysed across 11 populations in malaria endemic regions. We applied positional mapping, expression quantitative trait locus (eQTL), chromatin interaction mapping, and gene-based association analyses to identify candidate severe malaria resistance genes. We further applied rare variant analysis to raw GWAS datasets (*N* = 11,000) of three malaria endemic populations including Kenya, Malawi, and Gambia and performed various population genetic structures of the identified genes in the three populations and global populations. We performed network and pathway analyses to investigate their shared biological functions. Our functional mapping analysis identified 57 genes located in the known malaria genomic loci, while our gene-based GWAS analysis identified additional 125 genes across the genome. The identified genes were significantly enriched in malaria pathogenic pathways including multiple overlapping pathways in erythrocyte-related functions, blood coagulations, ion channels, adhesion molecules, membrane signalling elements, and neuronal systems. Our population genetic analysis revealed that the minor allele frequencies (MAF) of the single nucleotide polymorphisms (SNPs) residing in the identified genes are generally higher in the three malaria endemic populations compared to global populations. Overall, our results suggest that severe malaria resistance trait is attributed to multiple genes, highlighting the possibility of harnessing new malaria therapeutics that can simultaneously target multiple malaria protective host molecular pathways.

## Introduction

Malaria is still one of the global health problems with approximately 228 million cases and 405, 000 deaths in 2018 (WHO 2019). African countries disproportionately carry the global burden of malaria, accounting for 93 and 94% of cases and deaths, respectively (WHO 2019). *P*. *falciparum* malaria is still one of the leading causes of child mortality in endemic regions, particularly in sub-Saharan Africa. According to the World Health Organization (WHO), malaria killed about 285,000 under five children in 2016 (WHO 2018). About 10–20% of children who recover from severe malaria develop neurological sequalae and sub-optimal neuronal development (Egan et al., 2001). Severe malaria (SM) is defined as demonstration of asexual forms of the malaria parasites in the blood of a patient with a potentially fatal manifestation or complication of malaria in whom other diagnosis have been excluded (WHO 2014). The SM complications include rapid progression to severe malarial anaemia (SMA), hypoglycaemia, cerebral malaria (CM), acidosis, and death (WHO 2014).

*P*. *falciparum* has a complex life cycle that alternate between vertebrate and female *Anopheles mosquito.* During its blood meal, the infected mosquitoes inoculates the transmissive form of the parasite, the sporozoites, into human skin. From the skin, the sporozoites enter into the blood circulation or up-taken by the lymphatic system and invade liver (Miller et al., 2002). After maturation, the parasite buds off the hepatocytes and released into the circulation in the form of merozomes containing hundreds of thousands of merozoites that infect erythrocytes (Miller et al., 2002)*.* The erythrocyte stage also called blood stage lifecycle is a complex multi-step process that involves repeated invasion, growth, replication, and egress events (Cowman and Crabb 2006). The clinical symptoms of the SM have been linked to the blood stage life cycle (Cowman and Crabb 2006).

Even though SM is one of the commonest reasons for admission to hospital and is a major cause of hospital death in children aged 1–5 years in endemic areas, it constitutes only a small subset (1–2%) of the infected children as the majority of malaria infections are mild (Kevin et al., 1995). It has been shown that such clinical variations are partly attributable to human genetic factors (Damena et al., 2019; Damena and Chimusa 2020). Thus, a comprehensive understanding of the human genetic causes of variation in malaria clinical outcomes may potentially provide clues to design new intervention strategies such as therapeutics and vaccines (Kwiatkowski 2005; Teo, Small, and Kwiatkowski 2013).

Aiming at shedding more light to the genetic basis of severe *P*. *falciparum* malaria, several genome-wide association studies (GWASs) have been conducted in diverse malaria endemic populations over the last decade (Jallow et al., 2010; Timmann et al., 2012; Band et al., 2015; Ravenhall et al., 2018; Malaria Genomic Epidemiology Network, 2019). The GWASs have replicated some of the well-known malaria resistance genomic risk loci including sickle cell (*HBB*) and *ABO* blood group loci and identified new variants in *ATP2B4* and Glycophorin regions. Due to the single-marker testing approach commonly used, the GWASs miss candidate variants with weak genetic effects (Chimusa et al., 2015). To address these problem, a number of gene-based and pathway-level statistical analytic methods have been developed and successfully implemented in complex disease studies (Leeuw et al., 2015; Lamparter et al., 2016; Watanabe et al., 2017; Yoon et al., 2018). These methods can improve the study power by aggregating the joint effects of weakly associated markers at gene and pathway levels (Dudbridge 2016).

Furthermore, the methods integrate functional information from advanced biological databases including the Genotype-Tissue Expression (GTEx), (The GTex Consortium 2015), Encyclopedia of DNA Elements (ENCODE) (Hoffman et al., 2013), Roadmap Epigenomics Project Roadmap Epigenomics Consortium, 2015) and chromatin interaction information (Schmitt et al., 2016) to identify and prioritize candidate genes. Owing to the fact that direct functional follow-up of several candidate causal variants and genes is expensive, application of computational method to prioritize genes and their respective biological pathways are proven to be useful in complex diseases studies (Watanabe et al., 2017).

Here, we implement several gene-set, pathway and network analytic methods on summary statistics of severe malaria GWAS from 17,000 individuals meta-analysed across 11 populations and systematically predicted plausible genes and pathways. We further performed rare variant analysis on raw GWAS dataset (*N* = ∼11,000) of Kenya, Gambia, and Malawi populations. Finally, we performed population genetic structure analysis of the identified genes in the three malaria endemic countries and across global populations. Established over the course of long co-evolution time, blood stage life cycle of the parasite constitutes the most extensive interplay between host and parasite genomes which leads to the clinical symptoms of SM. Therefore, our results suggest that severe malaria resistance is polygenic and attributed to multiple genes aggregated in pathogenic pathways linked to the erythrocyte stage lifecycle of *P*. *falciparum.*

## Materials and Methods

### Description of the Study Datasets

We accessed a previous severe malaria GWAS datasets (Band et al., 2015) (*N* = ∼11,000) of three African populations including Kenya, Gambia, and Malawi from European Phenome Genome Archive (EGA) following the standard data access protocols outlined in previous studies (Achidi et al., 2008; Parker et al., 2009). Children with severe malaria cases were recruited on admission to hospital using definitions outlined by the WHO: cerebral malaria (Blantyre coma score <3 in children or Glasgow coma score <11 in adults), severe malarial anaemia (haemoglobin <5 g/100 ml or haematocrit <15%), and other malaria-related symptoms (Trampuz et al., 2003). Control samples were obtained from representative of the ethnic groups of the cases or in some study sites from the local population (Achidi et al., 2008). The samples were genotyped on Illumina Omni 2.5Marray and QC filtered as described in (Jallow et al., 2010).

In addition to the genotype dataset, we obtained a set of severe malaria susceptibility GWAS summary statistics (*N* = 17,000) meta-analysed across 11 population in Africa, Oceania, and Asia from Malaria Genomic Epidemiology Network (2019). The dataset contained information on GWASs of individual study populations and their meta-analysis. We additionally accessed reference dataset from 1,000 Genomes Project (The 1000 Genomes Project Consortium, 2011) and African Genome Variation Project (AGVP) (Gurdasani et al., 2015) (Supplementary Table S3).

### Functional Mapping and Annotations

We used the meta-analysed malaria GWAS summary statistics (*N* = 17,000 samples, 17 million SNPs) across 11 populations (Malaria Genomic Epidemiology Network 2019) for functional mapping and annotations. We implemented FUMA (Watanabe et al., 2017), a pipeline that determines genomic risk loci and prioritize potential causal genes by incorporating information from multiple sources including GTEx (The GTex Consortium 2015), Encyclopedia of DNA Elements (ENCODE) (Hoffman et al., 2013), Roadmap Epigenomics Project (Roadmap Epigenomics Consortium 2015), and chromatin interaction information (Schmitt et al., 2016).

Default settings of FUMA were used to determine the risk loci and independent significant SNPs from the malaria resistance GWAS summary statistics data. More specifically, independent significant SNPs are independent from each other for r2 < 0.6 and independent lead SNPs are independent from each other for r2 < 0.1, based on a pre-calculated LD structure using the African reference population of 1,000 Genomes version 3 which contain populations including GWD, MSL, ESN, YRI, and LWK (Watanabe et al., 2017). We then implemented three gene mapping strategies including positional mapping, expression Quantitative Trait Locus (eQTL) mapping, and chromatin interaction implemented in FUMA (Watanabe et al., 2017). Positional mapping was performed by ANNOVAR tool (Wang, Li, and Hakonarson 2010) using Ensembl (build 85; http://www.ensembl.org/) dataset. A maximum distance of 10 kb window size upstream and downstream was used to map SNPs to genes. SNPs filtering was carried out based on CADD score (Kircher et al., 2014), RegulomeDB score (Boyle et al., 2012), and 15-core chromatin state (Ernst and Kellis 2012).

eQTL mapping was performed for genes within 1 Mb of the most significant variant using datasets that contain eQTL information related to severe malaria such as brain and blood in FUMA software using default setting. These include PsychENCODE (Gulden et al., 2018), GTExv8 (Guerini, Pan, and Carafoli 2003), BRAINEAC (Ramasamy et al., 2014), DICE (Schmiedel et al., 2018), eQTLGen (Võsa et al., 2018), Blood eQTL browser (Zhernakova et al., 2016), and scRNA_eQTLs (Wijst et al., 2018). Chromatin interaction mapping was performed using datasets including GSE87112 (Schmitt et al., 2016), Hi-C loops (Giusti-Rodriguez et al., 2019; Huckins et al., 2019), PsychENCODE (Gulden et al., 2018), and FANTOM5 (Andersson et al., 2014).

To gain insights into the biological functions of the prioritized genes, we performed gene enrichment analysis using a hypergeometric test in which gene-sets obtained from MsigDB (Liberzon et al., 2011) and WikiPathways (Kutmon et al., 2016) were used as background genes. We further tested differential gene expression values on 54 tissues obtained from the GTEx (Guerini, Pan, and Carafoli 2003) as described in FUMA (Watanabe et al., 2017).

### Gene-Based Genome-Wide Association Analysis

Considering the polygenic nature of severe malaria susceptibility trait (Damena and Chimusa 2020), we applied Pascal (Lamparter et al., 2016), a gene-based GWAS analysis. Unlike the FUMA method which only identifies genes encoded by GWAS significant SNPs, Pascal method aggregates SNPs with modest effects and yields score for the corresponding genes. Briefly, we applied sum of chi-squared statistics (SOCS) analysis to the GWAS summary statistics of SM to compute the corresponding gene scores (*p*-values) using default settings of the Pascal software (Lamparter et al., 2016). LD information for estimation of correlation structure was obtained from African dataset in 1,000 G phase 3 (The 1000 Genomes Project Consortium 2011). We further categorized the prioritized genes into different functional groups using DAVID tools (Jiao et al., 2012). Significant genes after Bonferroni corrections were subjected to differential gene expression analysis implemented in FUMA software using 54 tissues obtained from the GTEx. Pathway scores were computed by combining the scores of genes that belong to the same gene-set using default parameter of the Pascal software (Lamparter et al., 2016).

### Gene Burden and Rare-Variants Association Analysis

Given that the GWAS assumption is based on common variant common disease hypothesis, GWAS approach always miss potential association signal from rare variants. To examine the contribution of rare variants, we applied optimal unified sequence kernel association test (SKAT-O) (Ionita-laza et al., 2013), which combines burden and variance-component analyses to the GWAS dataset of Gambia, Kenya, and Malawi populations. Briefly, we aligned the VCF files including Gambia (*N* = 4,920 samples, 1.6 million SNPs), Malawi (*N* = 2,560 samples, 1.6 million SNPs), and Kenya (*N* = 3,143 samples, 1.6 million SNPs) to GWAS dataset to 1,000 Genome v-3 reference haplotypes using Genotype Harmonizer (Deelen et al., 2014) and removed SNPs with position and strand mismatches and phased using SHAPEITv2 (Delaneau et al., 2013). We performed imputation using impute 2 (Howie et al., 2009) and obtained ∼20 million from each population. After removal of SNPs with low genotype rate and imputation accuracy, we retained ∼15,000 SNPs in each population. We then applied SKAT-O test to the quality filtered data following the procedure outlined in SKAT package (Ionita-laza et al., 2013).

### Network Analysis

To investigate the functional interactions between all the candidate malaria resistance candidate genes identified by FUMA and Pascal methods, we implemented network analysis. Briefly, we obtained functional interaction network of all the identified candidate malaria resistance genes using Multiple Association Network Integration Algorithm (geneMANIA) tool (Mostafavi et al., 2008). Using this information, we computed network parameters including degree, betweenness, and closeness centrality metrics to evaluate the topology of nodes (genes) and edges (interactions) in the network using networkX (Hagberg 2008) and R igraph packages (Csardi and Tamas, 2006). We excluded genes in the network which had betweenness score of zero and those with degree less than 3. We used the lowest betweenness and degree centrality score as a threshold to further filter the gene list to elucidate both provincial and connector hubs. From the analysis, we defined degree, betweenness, and closeness threshold of 17, 1,399, and 0.21502, respectively, to identify hubs in the network.

Closeness measures the average distance from the node to all other nodes in the network, indicating which nodes represent a greater “risk” (maximally close with lowest sum of edge weights) for eliciting other nodes. Betweenness measures the number of times that a node lies on the shortest path between two other nodes, indicating which nodes serve as a “hub” between other nodes (Csardi and Tamas, 2006). The degree of a node is described as the number of direct connections it has with other nodes within the network. Low degree nodes usually connect to nodes within their local community, whereas high degree nodes usually extend to the neighbouring community. Using the centrality scores, we quantified node centrality to identify hub genes by investigating the contribution of the edges and the weight of the edges towards node centrality. The hubs genes make strong contributions to the subnetwork and/or global network integrity. Connector hubs and provincial hubs refers to nodes that link other nodes across different communities and local communities, respectively.

### Population Genetic Structure of Malaria Resistance Candidate Genes

We performed the population genetic structure of the identified genes in malaria endemic populations (Kenya, Gambia, and Malawi) and global populations of 20 ethnic groups obtained from African Genome Variation Project (AGVP) (Gurdasani et al., 2015). We merged the quality filtered GWAS datasets of the three malaria endemic populations using PLINK software (Purcell et al., 2007). We performed basic quality control on both the merged malaria population dataset and AGVP dataset using PLINK software. We removed structural variants and ambiguous SNPs, removed SNPs with MAF below 0.01, deviate from Hardy-Weinberg at *p*-value below 0.01 and SNP missingness proportion greater than 0.02 (Marees et al., 2018).

We mapped SNPs in dbSNP database to the identified candidate genes using custom python script. We extracted the mapped SNPs from our datasets (Malaria GWAS and AGVP datasets) and retained for the downstream analyses.

We then partitioned the datasets into a total of 23 different Ethnic groups (20 from AGVP and three from malaria endemic populations) based on population or country label information. We clustered the merged malaria GWAS dataset into sub-regions/populations using smartpca software (Patterson, Price, and Reich 2006). To understand the frequency spectrum of the SNPS residing in the identified genes, we created different minor allele frequency (MAF) bins of all the SNPs mapped to our candidate genes for each ethnic group. We repeated the same analysis separately for each gene to obtain gene-specific MAF. We finally computed proportion of pathogenic SNPs contained in each of the candidate gene using ANNOVAR software (Wang, Li, and Hakonarson 2010).

## Results

### Functional Mapping and Annotations

**FIGURE 1:**
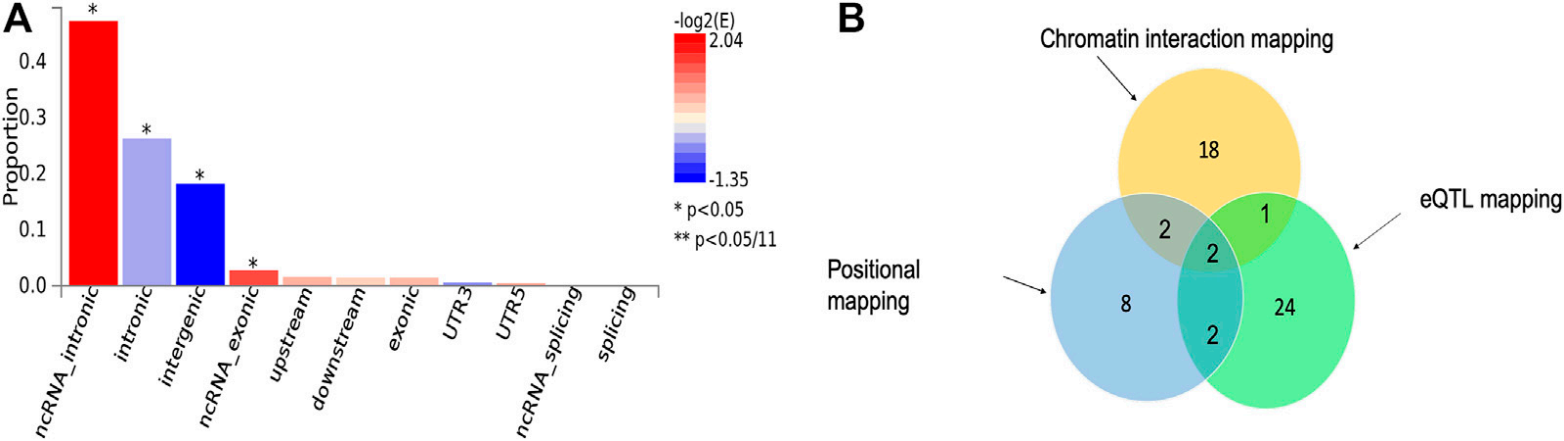
**(A)** Proportion of malaria resistance GWAS SNPs in different genomic annotation categories. **(B)** The number of genes identified by each of the three functional mapping strategies including positional mapping, eQTL, and chromatin interactions. The intersection sections of the circles depict the number overlapped genes between the respective mapping strategies.

We applied three functional mapping strategies implemented in FUMA (Watanabe et al., 2017) to the severe malaria GWAS summary statistics (see *Materials and Methods*). We identified 19 lead SNPs out of 69 significant SNPs across six genomic loci using default settings of the software (Supplementary Datas S1–S3). These SNPs were significantly enriched in ncRNA-intronic, intronic, intergenic, and ncRNA-exonic regions (Figure 1A).

Our functional mapping strategies yielded a total of 57 protein-coding genes (Table 1; Supplementary Data S4). These include 29, 23, and 14 genes identified by eQTL mapping, chromatin interaction mapping, and positional mapping, respectively (Figure 1B). Two genes including *ATP2B4* and *HBD* were identified by all the three gene mapping strategies, while five genes including *GYPB*, *HBG2*, *TRIM6-TRIM34*, *OR51F2*, and *TRIM68* were predicted by two of the three mapping strategies (Supplementary Data S4).

**TABLE 1:** Fifty-seven severe malaria resistance candidate genes identified by eQTL mapping, chromatin interaction mapping, and positional mapping strategies implemented in FUMA.

Genes -ensg	Symbols	Chr	Cytoband	Start	End	Biotype	Independent significant SNPs
ENSG00000160323	ADAMTS13	9	q34.2	136279478	136324508	Protein coding	rs8176751; rs687621
ENSG00000197859	ADAMTSL2	9	q34.2	136397286	136440641	Protein coding	rs8176751
ENSG00000179674	ARL14	3	q25.33	160394948	160396233	Protein coding	rs116423146
ENSG00000058668	ATP2B4	1	q32.1	203595689	203713209	Protein coding	rs4951370
ENSG00000169255	B3GALNT1	3	q26.1	160801671	160823172	Protein coding	rs116423146
ENSG00000159388	BTG2	1	q32.1	203274619	203278730	Protein coding	rs4951370
ENSG00000110148	CCKBR	11	p15.4	6280966	6293357	Protein coding	rs113892119:rs28576676
ENSG00000133063	CHIT1	1	q32.1	203181955	203242769	Protein coding	rs4951370
ENSG00000113758	DBN1	5	q35.3	176883609	176901402	Protein coding	rs687621
ENSG00000122176	FMOD	1	q32.1	203309756	203320617	Protein coding	rs4951370
ENSG00000183090	FREM3	4	q31.21	144498455	144621828	Protein coding	rs201510180
ENSG00000109458	GAB1	4	q31.21	144257915	144395721	Protein coding	rs111374053
ENSG00000148288	GBGT1	9	q34.2	136028340	136039332	Protein coding	rs687621
ENSG00000250361	GYPB	4	q31.21	144917257	145061844	Protein coding	rs201510180
ENSG00000197465	GYPE	4	q31.21	144792020	144826716	Protein coding	rs34330779
ENSG00000244734	HBB	11	p15.4	5246694	5250625	Protein coding	rs334
ENSG00000223609	HBD	11	p15.4	5253908	5256600	Protein coding	rs334; rs4290259; rs79681613; rs113892119:rs28576676
ENSG00000213931	HBE1	11	p15.4	5289582	5526847	Protein coding	rs145843585
ENSG00000213934	HBG1	11	p15.4	5269313	5271122	Protein coding	rs7927066
ENSG00000196565	HBG2	11	p15.4	5274420	5667019	Protein coding	rs145843585; rs183322782; rs148179286; rs7927066; rs11037724
ENSG00000203813	HIST1H3H	6	p22	27777842	27778314	Protein coding	rs8176751
ENSG00000122188	LAX1	1	q32.1	203734304	203745361	Protein coding	rs4951370
ENSG00000148297	MED22	9	q34.2	136205160	136214986	Protein coding	rs8176751; rs687621
ENSG00000108960	MMD	17	q22	53469974	53499353	Protein coding	rs8176751
ENSG00000167346	MMP26	11	p15.4	4726157	5013659	Protein coding	rs141862673; rs145429724
ENSG00000169251	NMD3	3	q26.1	160822484	160971320	Protein coding	rs116423146
ENSG00000184881	OR51B2	11	p15.4	5344541	5345582	Protein coding	rs145843585
ENSG00000176925	OR51F2	11	p15.4	4842551	4843686	Protein coding	rs141862673; rs145429724; rs113892119:rs28576676
ENSG00000176798	OR51L1	11	p15.4	5020213	5021160	Protein coding	rs113892119:rs28576676
ENSG00000182070	OR52A1	11	p15.4	5172239	5207612	Protein coding	rs116780407
ENSG00000228474	OST4	2	p23.3	27293340	27294641	Protein coding	rs8176751
ENSG00000142657	PGD	1	p36.22	10458649	10480201	Protein coding	rs687621
ENSG00000143850	PLEKHA6	1	q32.1	204187979	204346793	Protein coding	rs4951370
ENSG00000163590	PPM1L	3	q25.33	160473390	160796695	Protein coding	rs116423146
ENSG00000188783	PRELP	1	q32.1	203444956	203460480	Protein coding	rs4951370
ENSG00000170955	PRKCDBP	11	p15.4	6340176	6341877	Protein coding	rs28576676
ENSG00000160271	RALGDS	9	q34.2	135973107	136039301	Protein coding	rs8176751; rs687621
ENSG00000148300	REXO4	9	q34.2	136271186	136283164	Protein coding	rs8176751
ENSG00000080345	RIF1	2	q23.3	152266397	152364527	Protein coding	rs8176751
ENSG00000170153	RNF150	4	q31.21	141780961	142134031	Protein coding	rs111374053
ENSG00000136193	SCRN1	7	p14.3	29959719	30029905	Protein coding	rs687621
ENSG00000160326	SLC2A6	9	q34.2	136336217	136344259	Protein coding	rs8176719; rs687621
ENSG00000196542	SPTSSB	3	q26.1	161062580	161090668	Protein coding	rs116423146
ENSG00000148290	SURF1	9	q34.2	136218610	136223552	Protein coding	rs8176751; rs687621
ENSG00000148291	SURF2	9	q34.2	136223428	136228045	Protein coding	rs8176751
ENSG00000148248	SURF4	9	q34.2	136228325	136242970	Protein coding	rs8176751
ENSG00000148296	SURF6	9	q34.2	136197552	136203235	Protein coding	rs8176751; rs687621
ENSG00000196628	TCF4	18	q21.2	52889562	53332018	Protein coding	rs687621
ENSG00000132109	TRIM21	11	p15.4	4406127	4414926	Protein coding	rs28576676
ENSG00000132274	TRIM22	11	p15.4	5710919	5758319	Protein coding	rs28576676
ENSG00000258659	TRIM34	11	p15.4	5640994	5665628	Protein coding	rs183322782; rs148179286
ENSG00000213186	TRIM59	3	q25.33	160150233	160203561	Protein coding	rs116423146
ENSG00000121236	TRIM6	11	p15.4	5617339	5634188	Protein coding	rs28576676
ENSG00000258588	TRIM6-TRIM34	11	p15.4	5617955	5665628	Protein coding	rs183322782; rs148179286; rs28576676
ENSG00000167333	TRIM68	11	p15.4	4619902	4629489	Protein coding	rs10837488; rs4290259:rs113892119:rs28576676
ENSG00000175518	UBQLNL	11	p15.4	5535623	5537935	Protein coding	rs11037724
ENSG00000109445	ZNF330	4	q31.21	142142041	142155851	Protein coding	rs111374053

**FIGURE 2:**
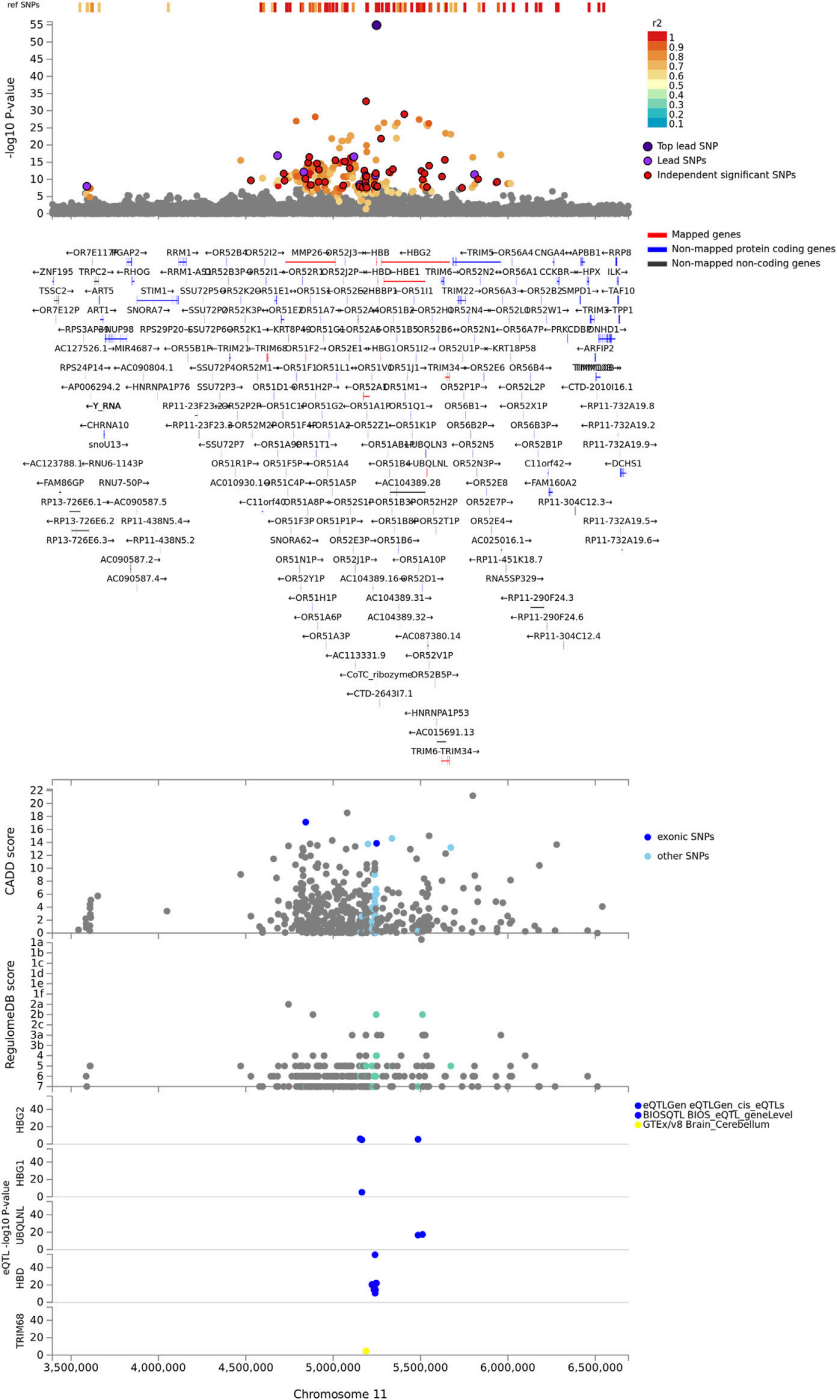
Regional plot of severe malaria susceptibility GWAS locus on chromosome 11. Non-GWAS-tagged SNPs are shown at the top of the plot as rectangles since they do not have a *p*-value from the GWAS. Prioritized genes are highlighted in red. eQTLs are plotted per gene and coloured based on tissue types. CADD score, RegulomeDB score, and eQTLs, SNPs which are not mapped to any gene are coloured grey.

The identified genes were enriched in five cytogenic positions including chr11p15 (*p* = 2.65e-18), chr9q34 (*p* = 4.63e-9), chr4p31 (*p* = 4.6e-8), chr1q32 (*p* = 2.6e-7), and chr3q26 (*p* = 7.97e-4) (Supplementary Table S1). We noted that the majority (33%) of the identified genes were clustered on chr11p15 (Figure 2). These include beta globin gene cluster: *HBB*, *HBD*, *HBG1*, *HBG2*, and *HBE1*; Tripartite motif-containing (*TRIM*) family genes including *TRIM68* and *TRIM21*; and genes involved in olfactory receptors and G protein-coupled signalling (GPCR) such as *CCKR OR51F2* and *OR51L* (Supplementary Table S1)*.* About two-thirds (13/19) of the genes in this locus are in eQTL and chromatin interactions (Supplementary Data S4; Supplementary Figure S1).

All the implicated genes in chr9q34 locus are located outside the genomic risk locus and were identified by eQTL mapping (Supplementary Data S4; Supplementary Figure S2). These include surfeit gene cluster such as *SURF2*, *SURF4*, *MED22*, and *SURF6*; a metalloprotease gene, *ADAMTS13*; and a gene encoding *ORS* blood group system coding gene (*GBGT1*). In the remaining enriched cytogenic positions, the known genes including *ATP2B4* (chr1q32) and *FREM3*, *GYPE*, and *GYPB* (chr4p31) were replicated. Other notable genes include *BTG2*, a tumour suppressor gene on chr1q32, and *B3GALNT1* on chr3q26 (Supplementary Data S4).

### Candidate Genes Identified by Gene-Based GWAS Analysis Using Pascal Method

Taking the polygenic nature of severe malaria resistance trait into consideration (Damena and Chimusa 2020), we applied a pathway scoring algorithm (Pascal) (Lamparter et al., 2016) method can capture polygenic effects at the gene level (see *Materials and Methods*). The Pascal analysis replicated 13 genes that were identified by FUMA (Supplementary Table S2) and identified additional 125 genes across the genome (Supplementary Data S5). The genes with top scores outside genomic risk loci include *CSMD1* (*p* = 1.58e-12) on chr8p23.2 and *RBFOX1* (*p* = 9.76e-11) on chr16p13.3. *CSMD1* is an important regulator of complement activation and inflammation (Sun et al., 2001; Lee et al., 2019), while *RBFOX1* encodes for an mRNA-splicing factor linked to autism spectrum disorders (Hamada et al., 2016). A previous study in Tanzanian population reported association of variants in *RBFOX* gene with SM (Ravenhall et al., 2018).

Other important genes identified by gene-based GWASs include neural adhesion molecules *CNTN4* (*p* = 3.88e-9) on chr3p26.3-p26.2 which has been linked to autism spectrum disorders (Fernandez et al., 2004), *PCSK5*(*p* = 2.88e-11) on chr9q21.13, and *CDH13* (*p* = 4.19e-8) on chr16q23.3) and *TMEM132* (*p* = 2.18e-8) on chr17q12 (Supplementary Data S5).

Furthermore, protein kinases including *FLT4* (*p* = 9.96e-8) on chr5q35.3, *PTPRT* (*p* = 4.92e-7) on chr20q12-q13, and *PRKG1* (*p* = 1.2e-6) on chr10q11.2-q21.1 were among the genes with top scores (Supplementary Data S5). *PTPRT* is a tyrosine phosphatase receptor involved in STAT3 pathway and was recently reported to be associated with mild malaria susceptibility in Benin populations (Milet et al., 2019). *PRKG1* is a cyclic guanosine monophosphate (GMP) dependent protein kinase which plays important roles in relaxation of vascular smooth muscle and inhibition of platelet aggregation (Mrozek et al., 2003). *FLT4* acts as a cell-surface receptor for vascular endothelial growth factor C (VEGFC) and vascular endothelial growth factor D (VEGFD), and plays an essential role in the development of the vascular network (Aprelikova et al., 1992). It has been shown that VEGF is expressed in the brain tissues and reported to play protective during CM (Yeo et al., 2008).

### Gene-Based Rare Variant Association

Because rare variants are known to play role in the variation of most complex traits, we applied optimal unified sequence kernel association test (SKAT-O), which combines burden and variance-component analyses (Ionita-laza et al., 2013), to the raw genotype GWAS dataset of Gambia, Kenya, and Malawi populations (see Materials and Methods). The SKAT-O analysis identified a total of six and nine nominally significant genes in Gambia and Malawi populations, respectively. These include nine long intergenic non-protein coding RNAs (LincRNAs), *MIR4282*, *GLYR1*, *NDNF*, *EPB41L2*, *ATP8A1*, and *WASF3* (Supplementary Table S3). However, none of these genes were significant after correction for multiple testing.

### Functional Networks and Subnetworks of Severe Malaria Resistance Candidate Genes

**FIGURE 3:**
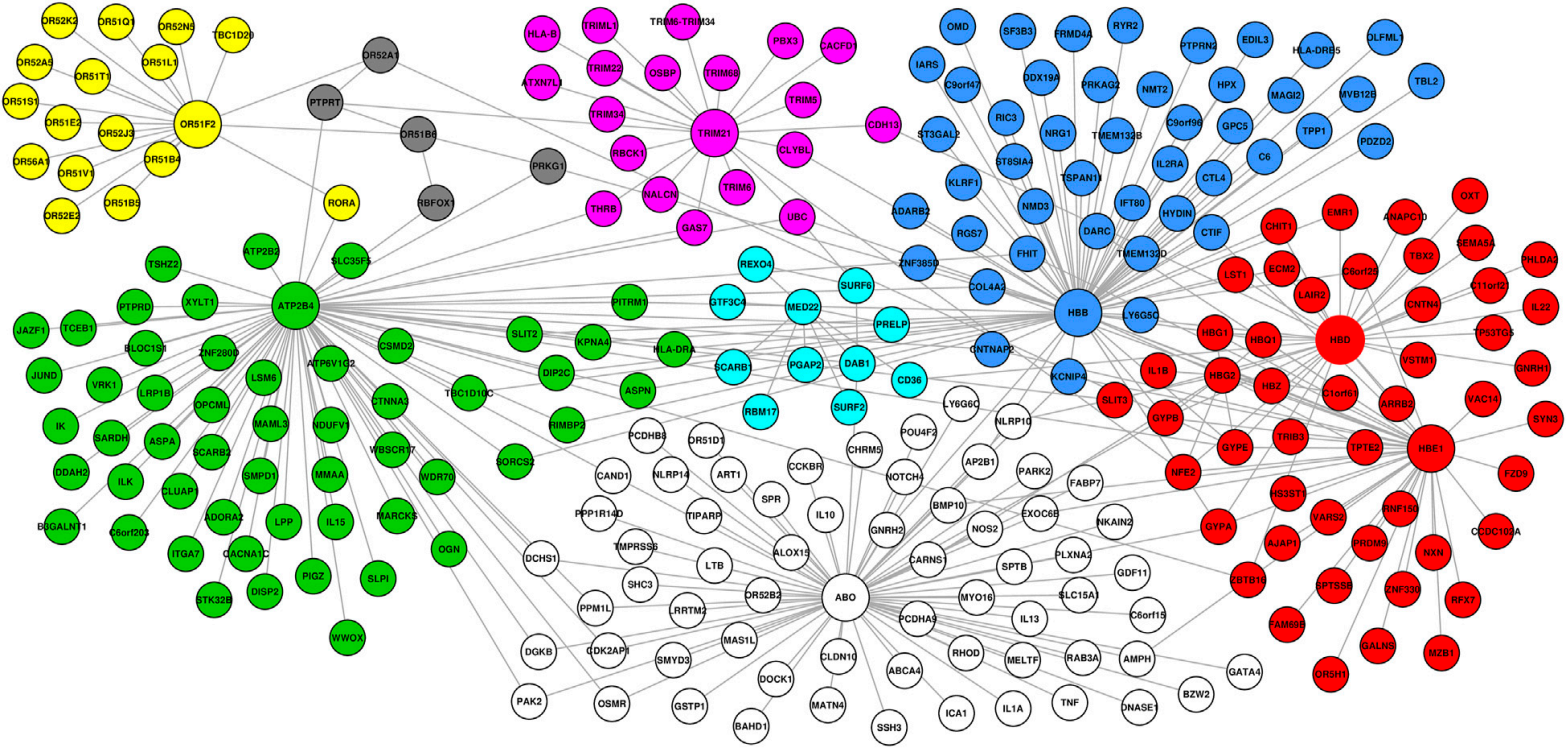
Network generated from predominant severe malaria protective candidate genes, comprising 351 interactions between 268 nodes. Topology analysis identified ABO, HBB, HBD, HBE1, and ATP2B4 as highly influential connector hub genes influencing at least two subnetworks/communities, while TRIM21 and OR5F2 constituted independent communities. MED22 and OR551B6 constituted provincial hub genes.

To investigate the functional interaction between all the candidate SM resistance candidate genes identified in this study, we implemented network analysis (see Materials and Methods). Our global network generated 351 functional interactions between 268 genes. Topology analysis identified *ABO*, *HBB*, *HBD*, *HBE1*, and *ATP2B4* as highly influential connector hub genes influencing at least two subnetworks/communities, while *TRIM21* and *OR5F2* constituted independent communities. *MED22* and *OR551B6* constituted provincial hub genes (Figure 3).

### Molecular Functions of Genes in Malaria Risk Loci Identified by FUMA Method

To test whether the genes predicted by the three functional mapping strategies overlapped in functional gene sets and pathways, we conducted gene enrichment analysis implemented in FUMA (Watanabe et al., 2017) using MsigDBc5 (Liberzon et al., 2011) gensets as background (see Materials and Methods). The gene enrichment analysis identified several shared biological functions linked to erythrocyte-related pathways including three gene ontology (GO) cellular components, eight GO molecular functions, and 14 GO biological processes (Table 2). The implicated cellular components include haptoglobin-haemoglobin complex (*p* = 7.6e-8), haemoglobin-complex (*p* = 7.63e-8), and cytosolic-part (*p* = 6.73e-3). The enriched molecular functions include haptoglobin binding (*p* = 4.87e-8), oxygen carrier activity (3.8e-7), oxygen binding (*p* = 4.87e-5), and other activities related to haemoglobin functions. The shared biological activities include oxygen transport (*p* = 4.22e-6), gas transport (*p* = 4.87e-6), hydrogen peroxide catabolism (*p* = 9.08e-5), protein hetero-oligomerization (*p* = 2.86e-3), protein complex-oligomerization (*p* = 4.23e-3), interferon gamma mediated signalling pathways (*p* = 3.09e-2), and blood coagulation (*p* = 3.09e-2). Our differential expression analysis of genes identified by FUMA method did not yield significant enrichments.

**TABLE 2:** Gene enrichment results of functionally annotated genes in malaria genomic risk loci identified by FUMA method.

GO terms	Geneset	N. genes	N. enriched genes	*p*-value	Adjusted *p*-value	Genes
Cellular components	Haptoglobin-haemoglobin complex	11	5	8.91e-11	7.63e-8	HBB, HBD, HBG1, HBG2, HBE1
	Haemoglobin complex	12	5	1.52e-10	7.63e-8	HBB, HBD, HBG1, HBG2, HBE1
	Cytosolic part	239	7	6.73e-6	2.25e-3	HBB, HBD, HBG1, HBG2, HBE1, DBN1, SURF6
Biological functions	Haptoglobin binding	10	5	4.87e-11	8.02e-8	HBB, HBD, HBG1, HBG2, HBE1
	Oxygen carrier activity	14	5	3.84e-10	3.16e-7	HBB, HBD, HBG1, HBG2, HBE1
	Oxygen binding	36	5	6.87e-8	3.77e-5	HBB, HBD, HBG1, HBG2, HBE1
	Molecular carrier activity	41	5	1.35e-7	5.56e-5	HBB, HBD, HBG1, HBG2, HBE1
	Oxidoreductase activity acting on peroxide as acceptor	56	5	6.66e-7	2.19e-4	HBB, HBD, HBG1, HBG2, HBE1
	Haemoglobin binding	7	3	8.69e-7	2.38e-4	HBB, HBD, HBE1
	Antioxidant activity	85	5	5.35e-6	1.26e-3	HBB, HBD, HBG1, HBG2, HBE1
	Tetrapyrrole binding	136	5	5.23e-5	1.08e-2	HBB, HBD, HBG1, HBG2, HBE1
Biological processes	Oxygen transport	15	5	5.74e-10	4.22e-6	HBB, HBD, HBG1, HBG2, HBE1
	Gas transport	19	5	2.20e-9	8.10e-6	HBB, HBD, HBG1, HBG2, HBE1
	Hydrogen peroxide catabolic process	32	5	3.71e-8	9.08e-5	HBB, HBD, HBG1, HBG2, HBE1
	Antibiotic catabolic process	50	5	3.74e-7	6.88e-4	HBB, HBD, HBG1, HBG2, HBE1
	Drug catabolic process	108	6	8.05e-7	1.18e-3	CHIT1, HBB, HBD, HBG1, HBG2, HBE1
	Cofactor catabolic process	66	5	1.52e-6	1.87e-3	HBB, HBD, HBG1, HBG2, HBE1
	Protein hetero-oligomerization	133	6	2.73e-6	2.86e-3	HBB, HBD, HBG1, HBG2, HBE1, HIST1H3H
	Protein complex oligomerization	551	10	4.60e-6	4.23e-3	TRIM21, HBB, HBD, HBG1, HBG2, HBE1, TRIM6, TRIM34, TRIM22, HIST1H3H
	Antibiotic metabolic process	91	5	7.49e-6	6.11e-3	HBB, HBD, HBG1, HBG2, HBE1
	Cellular detoxification	107	5	1.65e-5	1.21e-2	HBB, HBD, HBG1, HBG2, HBE1
	Protein trimerization	54	4	2.00e-5	1.34e-2	TRIM21, TRIM6, TRIM34, TRIM22
	Detoxification	122	5	3.11e-5	1.91e-2	HBB, HBD, HBG1, HBG2, HBE1
	Interferon gamma mediated signalling pathway	70	4	5.60e-5	3.09e-2	TRIM21, TRIM68, TRIM34, TRIM22
	Coagulation	335	7	5.89e-5	3.09e-2	HBB, HBD, HBG1, HBG2, HBE1, HIST1H3H, ADAMTS13
	Oxygen transport	15	5	5.74e-10	4.22e-6	HBB, HBD, HBG1, HBG2, HBE1

### Molecular Functions of Candidate Genes Identified by Gene-Based GWAS Analysis Using Pascal Method

In addition to genes within SM genomic risk loci, we performed functional analysis and pathway analysis for the genes identified by the gene-based GWAS using Database for Annotation, Visualization and Integrated Discovery (DAVID) method (Jiao et al., 2012) and Pascal (Lamparter et al., 2016), respectively (see Materials and Methods).

**FIGURE 4:**
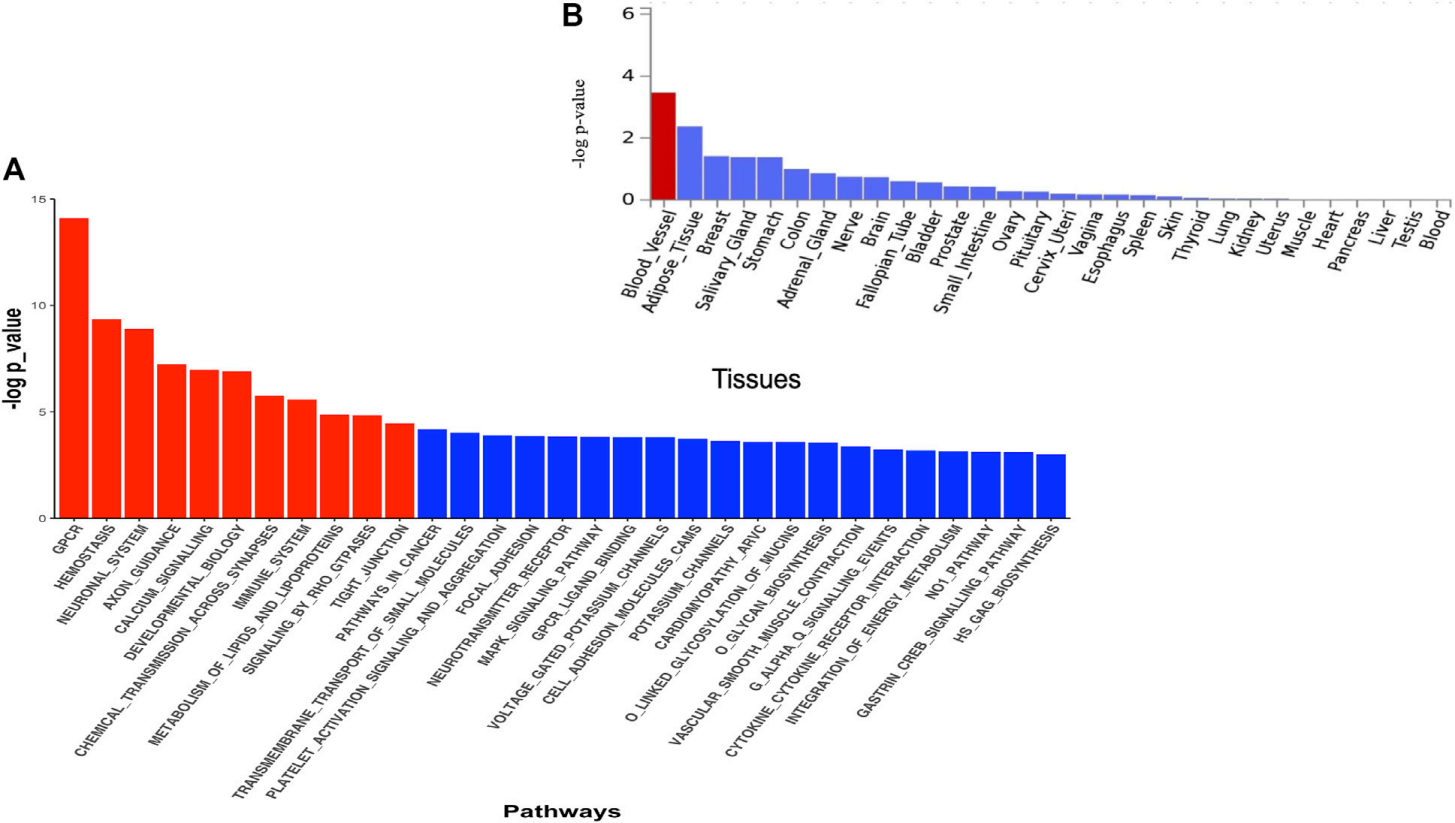
**(A)** Pathway scores obtained from pascal analysis. Significant pathways at Bonferroni corrected *p*-value ≤ 0.05 are coloured in red. **(B)** Tissue specific gene expression and shared biological functions of genes identified by pascal in GTEx v8 30 general tissue types. Input genes were tested against each of the precomputed differentially expressed sets using the hypergeometric test. Significant enrichment at Bonferroni corrected *p*-value ≤ 0.05 are coloured in red.

The DAVID analysis yielded eight functional categories, the majority of which are linked to malaria pathogenesis (Table 3) including GPCR signalling, membrane/transmembrane proteins, Na+/K+ transporting ATPases, cell adhesions, haemoglobin related functions, calcium signalling, and actin binding activities. The Pascal analysis replicated pathways including haemostasis (*p* = 4.52e-10), G protein-coupled receptor signalling (*p* = 7.88e-15), and calcium signalling (*p* = 1.10e-7) (Figure 4A). Additional pathways including neuronal system (*p* = 1.25 e-9), axon guidance (*p* = 5.93e-8), chemical transmission across synapses (*p* = 1.75 e-7), immune system (*p* = 2.61e-6), signalling by Rho GTPase (*p* = 1.45 e-5), and tight junction (*p* = 3.57 e-5) were identified by this method. Furthermore, differential expression of genes implicated by Pascal method showed significant enrichment in blood vessels (Figure 4B).

**TABLE 3:** Functional categories of genes identified by gene-based GWAS analysis grouped using DAVID method.

Functional group	Genes	Enrichment score
GPCR signalling pathways and olfactory receptions	OR51B6, FZD10, TMEM132C, OR51V1, OR51B2, OR52K2, SORCS2, OR56A1, OR52B4, TMEM132D, OR51E2, OR51T1, OR51B4, OR51B5, VSTM1, OR51F2, THSD7B	3.15
Transmembrane protein and Na+/K+ transporting ATPase	TSPAN11, VSTM1, TMEM132D, SURF4, NKAIN2, TMEM132C, EVC, SLC35F3	3.07
Tyrosine phosphatase, tyrosine kinase, cell adhesion molecule-like	OPCML, PTPRD, PTPRT, CNTN5, FLT4, NTM, CNTN4, PTPRN2, VSTM1, PTPRS	2.51
Haemoglobin related activities	HBG2, HBE1, HBB, HBD	2.42
Sodium leak and potassium channel interacting protein	KCNIP1, NALCN, KCNIP4, KCTD1	1.14
Zinc finger protein	SMYD3, TSHZ2, ZNF385B, ZNF385D	0.61
Actin binding LIM protein family and RAR-related orphan receptor A	RORA, THRB, GLIS3, ABLIM2	0.45
Calcium/calmodulin-dependent protein kinase, cGMP-dependent kinase, and fms-related tyrosine kinase	PRKG1, CAMK1D, FLT4, VRK1	0.44

### Population Genetic Structure of Malaria Resistance Candidate Genes

To assess the levels of differentiation of the candidate genes, we performed population genetic structure analyses in malaria endemic populations (Kenya, Gambia, and Malawi) and global populations of 20 ethnic groups obtained from African Genome Variation Project (AGVP) (Gurdasani et al., 2015). We mapped a total of 14,106,476 of SNPs in dbSNP database to the identified candidate genes. Out of these, we retained a total of 15,675 SNPs and 93,5549 SNPs that are in the Malaria GWASs dataset (*N* = 10,578 samples) and AGVP (*N* = 4,932 samples) dataset, respectively. We partitioned these datasets into individual population and computed population structure analyses including PCA, MAF, and proportion of pathogenic SNPs residing in the candidate genes (see *Materials and Methods*).

**FIGURE 5:**
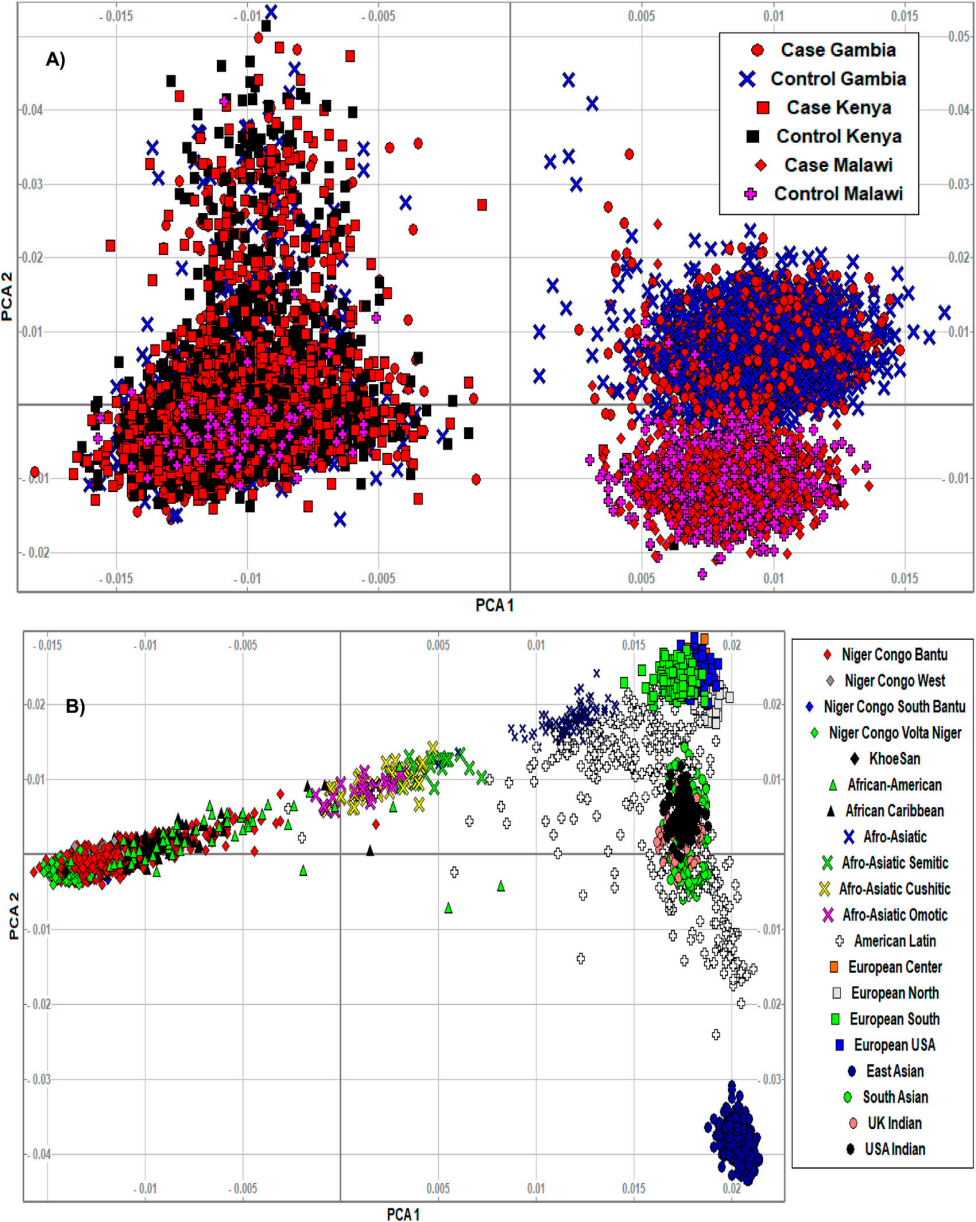
**(A)** We clustered the merged malaria GWAS dataset containing only SNPs residing in the identified malaria resistance candidate genes (*N* = 10,578 samples, 15,675 SNPs) using smartpca software. The populations were indicated by different colours and symbols. The PCA analysis effectively clustered the majority of the populations according to their ancestry, suggesting that the variants in the candidate genes are differentiated across ethnic background. The three populations and their case/control status were indicated by different colours and symbols. **(B)** We clustered the AGVP dataset containing only SNPs residing in the identified malaria resistance candidate genes (*N* = 4,932 samples, 93,5549 SNPs) into sub-regions/populations using smartpca software. The populations were indicated by different colours and symbols. The populations were clustered based on their geographical locations. However, the clusters in Malawi and Gambia slightly overlapped, likely because of the similar malaria endemicity in these population.

**FIGURE 6:**
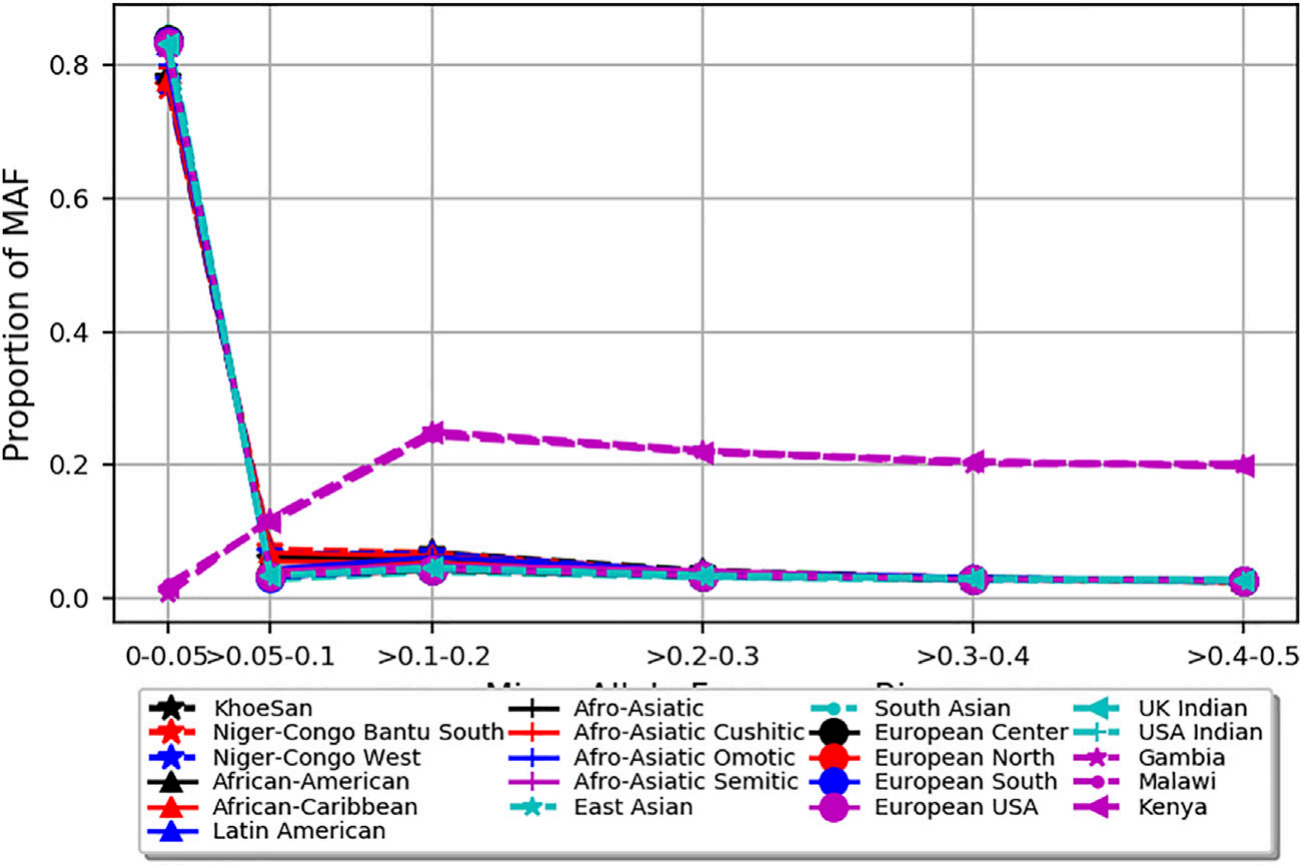
Depicts minor allele frequency (MAF) bins of SNPs mapped to severe malaria resistance candidate genes in three malaria endemic populations (Gambia, Malawi, and Kenya) and global populations composed of 20 ethnic groups. *Y*-axis represents allele frequency, and *X*-axis represents different MAF bins. Populations were represented by different colors and symbols.

The PCA analysis effectively clustered the majority of the populations according to their ancestry (Figure 5). This suggests that the SNPs residing in the candidate genes are differentiated across geographical locations and ethnic background. We also noted that the minor allele frequencies (MAF) of common SNPs (MAF > 0.05) in the candidate genes are generally higher in the three malaria populations compared to 20 ethnic groups (Figure 6; Supplementary Figure S3). We further observed that the proportion of pathogenic SNPs in a total of 18 genes is much higher in the three malaria endemic populations compared to other populations (Supplementary Datas S6, S7). These include *TRIM* family genes such as *TRIM21*, *TRIM22*, *TRIM68*, *TRIM6-TRIM34*, *and TRIM34* in which the pathogenic SNP proportion ranges from 13.3 to 25%, and olfactory receptors genes such as *OR51B4*, *OR51B6*, *OR51B2*, *OR56A1*, *OR51L1*, *OR52K2*, and *OR51E2* in which the pathogenic SNP proportion ranges from 27.3 to 100%.

## Discussion

In this study, we applied statistical functional analytic method to the largest ever severe malaria susceptibility GWAS dataset and identified the well-known malaria resistance loci and a number of novel genes that can guide future functional experiments. We noted that severe malaria resistance is attributed to multiple genes and pathways linked to malaria pathogenesis during blood stage life cycle of the parasite including merozoite invasion, parasite growth, cytoadherence, and signal transduction. The genes that were identified by our three mapping strategies might have equal importance; genes that were identified by positional mapping may act at protein level through structural changes, while the genes identified by eQTL and chromatin interactions exert their influences through quantitative changes at gene expression levels (Watanabe et al., 2017).

The fact that the functionally mapped genes are clustered on chr11p15 is consistent with our recent work in which we reported the disproportionate concentration of SNP-heritability on chr 11. This might reinforce the need for targeting this chromosome in the future severe malaria resistance studies (Damena and Chimusa 2020). In addition to the sickle trait gene (*HBB*), our mapping strategies identified other members of beta globin gene cluster that cause various forms of beta-thalassemia (*HBE1*, *HBD*, *HBG*, and *HBG2*). Our network analysis showed that all these genes constituted hub with which several other genes are connected, which reaffirm the importance of hemoglobinopathies in resistance against severe malaria. Hemoglobinopathies are believed to confer protection against severe malaria by suppressing the parasite growth and by mitigating associated pathogenic effects (Taylor, Cerami, and Fairhurst 2013). The proposed protective mechanisms of hemoglobinopathies against severe malaria have been extensively reviewed elsewhere (Hedrick 2011; Taylor, Cerami, and Fairhurst 2013).

In addition to the beta globin gene cluster, the Tripartite motif (*TRIM*) containing gene family (*TRIM68* and *TRIM21*) identified in this locus are known to play critical role in down regulating Toll-like receptors (TLR)- and Rig-like receptors (RLR)-induced responses and protect from autoimmune and inflammations (Mccarthy et al., 2014; Foss et al., 2019). Mal-adapted inflammatory reactions is one of the hall mark pathogenic pathways in severe malaria (Milner 2018; Moxon et al., 2020). The olfactory receptor super-family genes (*CCKR*, *OR51F2*, and *OR51L*) identified in this locus might be involved in G protein-coupled receptor (GPCR) signalling activities which is important in blood stage life cycle of *P*. *falciparum* (Mbengue, Yam, and Braun-breton 2012)*.* However, it is also possible that these genes were detected because of their abundance and close proximity to globin gene cluster (Alonso, Lo, and Izagirre 2006). We also noted that the genes in the *TRM* family and olfactory receptor super-family contain higher proportion of pathogenic SNPs in the three-malaria endemic population compared to the global populations. The majority of the well-known malaria protective genes have deleterious variants and were evolved under balancing selections.

Our eQTL mapping identified *ADAMTS13* gene on chr9q34 outside the malaria genomic risk locus which would not have been identified by the conventional SNP mapping approach (Watanabe et al., 2017). *ADAMTS13* is a zinc-containing metalloprotease enzyme that cleaves von Willebrand factor vWF, a large protein derived from endothelial surface and megakaryocytes which plays a crucial role in basic haemostasis (Sporn et al., 1989; Zheng, 2016). Following activation of endothelial cells, vWF is directly released into plasma and basement membrane or is stored in Weibel–Palade bodies (WPBs) from where it is released by regulated secretion to promote adhesion of platelets at the sites of vascular injury and facilitate vascular healing (Sporn et al., 1989). However, abnormal accumulations of vWF caused by deficiency of plasma *ADAMTS13* trigger intravascular platelet aggregation and micro thrombosis leading to a vascular disease, thrombotic thrombocytopenic purpura (TTP) (Zheng, 2016). Indeed, recent works have linked the platelet-mediated clumping of infected erythrocytes in microvasculature during cerebral malaria with increased level of VWF in plasma caused by mutations in *ADAMTS13* genes (Hollestelle et al., 2006; Kraisin et al., 2011; Adams et al., 2014)*.*

In the same genomic locus, our eQTL functional mapping identified surfeit gene cluster, metalloprotease genes linked to epithelial adhesions and blood coagulations. *SURF4* gene has been implicated in epithelial cell adhesion trait (Ahsan et al., 2017), *MED22* gene is linked to VWF factor/factor VIII level measurement (Sabater-Lleal et al., 2019), and *SURF6* has been implicated in epithelial ovarian cancer (Chen et al., 2014). Our network analysis showed that *MED22* forms a central hub to which the rest of surfeit gene clusters are connected. This may suggest the greater importance of *MED22* gene compared with other members of the cluster. In the remaining genomic risk loci, the well-known genes that play role in severe malaria resistance (Timmann et al., 2012; Band et al., 2015) including *ATP2B4* (chr1q32), *FREM3*, *GYPE*, and *GYPB* (chr4p31) were replicated and few additional genes were identified. The glycophorin gene clusters, *GYPA* and *GYPB*, encode the MNS blood group system and are host-erythrocyte receptors for *P. falciparum*, suggesting that polymorphisms in these genes play protective role by interfering with the invasion processes (Sun et al., 2001). *ATP2B4* variants may impair the parasite lifecycle in erythrocyte by affecting intracellular Calcium homeostasis (Tiffert et al., 2005; Malaria Genomic Epidemiology Network 2019).

Novel genes identified in these loci include *BTG2* on chr1q32 and *B3GALNT1* on chr3q26*. BTG2* is a tumour suppressor gene known to be linked to RBC related traits including MCHC level, RBC distribution, and reticulocyte count (Astle et al., 2017). *B3GALNT1* encodes globoside blood group system which is determined by P antigen (Hellberg, Poole, and Olsson 2002). Globoside/P antigen is the most abundant neutral glycolipid in the erythrocyte membrane and has been recognized as a cellular receptor for parvo-B19 virus (Kevin et al., 1994). Individuals lacking this receptor are resistant to parvo-B19 virus and uro-pathogenic *E*. *coli* infections (Lund et al., 1985; Kevin et al., 1994). This may suggest that variants in these genes might influence susceptibility to infectious diseases including malaria. However, further investigations are needed to establish the link between these genes and SM resistance.

We noted that the genes identified in malaria risk loci share cellular components including haptoglobin binding, haemoglobin complex, and cytosolic part and several overlapped molecular functions and biological processes linked with the blood stage life cycle of the parasite. *P. falciparum* spends most of its lifecycle within RBCs, where it undergoes multiple rounds of invasion, growth, replication, and egress, causing the signs and symptoms of malaria (Rowe et al., 2009; Moxon et al., 2020). The majority of the classical haemoglobin variants confer protection against severe malaria by restricting invasion process and intraerythrocytic growth of the parasite (Taylor, Cerami, and Fairhurst 2013). Haptoglobin is an acute phase glycoprotein present in human plasma. It forms stable complexes with extracellular haemoglobin that is released from lysed RBCs and thereby curtail the haemoglobin-induced oxidative tissue damage (Kristiansen et al., 2001).

*P*. *falciparum* ingest the host cell cytosol to obtain nutrients and space for growth in the RBCs (Francis, Sullivan, and Goldberg 1997). A recent study showed that the host cytosol uptake process is mediated by parasite’s protein called *VPS45* (Jonscher et al., 2019). The fact that the identified candidate genes in this study were enriched in the cytosol part of the cellular component might suggest that the variants of these genes might arrest the nutrient up-take of the parasites and thereby confer protection against their pathogenic effects. In addition to haemoglobin related functions, some of the candidate genes were enriched in pathways linked to malaria pathogenesis including blood coagulation related processes (Francischetti, Seydel, and Monteiro 2008; Sullivan et al., 2016) and interferon gamma mediated signalling pathways (Hunt et al., 2014; Tosevski et al., 2017). This may suggest that the host genetic factors might interfere with parasite development and its pathogenic effects at multiple levels to confer protection against the life-threatening form of malaria.

Our gene-based GWAS analysis replicated the well-known malaria resistance candidate genes in the genomic risk loci and identified several genes across the genome. Genes with top scores encode for different malaria relevant functions such as regulation of inflammation (*CSMD1*), neural adhesion (*CNTN4*, *PCSK5*, *CDH13*, *TMEM132*), vascular epithelial development (*FLT4*), and protein kinases (*PTPRT*, *PRKG1*). Functional analyses of these genes yielded functional categories linked to the blood stage lifecycle of the parasite and associated pathologies. The top enriched functions include GPCR signalling, membrane/transmembrane proteins (Na+/K+) transporting ATPases, sodium leak and potassium channel interacting proteins, cell adhesions molecules and calcium/calmodulin-dependent protein kinases (CDPKs), cell adhesion molecules, Rho GTPase activities, tight junction, neuronal system, and axon guidance.

CDPKs have crucial functions in calcium signalling at various stages of the parasite’s life cycle and is proposed to be one of the potential drug targets against malaria (Ghartey-kwansah et al., 2020). It has been shown that *P*. *falciparum* infection activates host signalling pathway involving protein kinase C (PKC) (Sicard et al., 2011). Similarly, host GPCR signalling pathways have been shown to play vital roles in invasion, intra-erythrocyte parasite development, and egress processes (Millholland et al., 2013; Brochet and Billker 2016), suggesting the existence of substantial interactions between host membrane/transmembrane signalling and parasite signalling elements which might mediate the disease severity. Further studies are needed to decouple the host-parasite interface of signal transduction and explore the potential target for new therapeutics.

Sodium leak and potassium channel interacting proteins (Na^+^/K^+^) transporting ATPases play critical role in maintaining electrochemical equilibrium in normal erythrocytes. However, upon invasion by trophoblast stage of the parasite, the ion pump-leak balance is perturbed, with increased leak rate and decreased pump rate resulting in a remarkable increase in (Na^+^) and decrease in (K^+^) in the erythrocyte cytosol (Pillai et al., 2013; Desai 2014). This results in formation of a new permeability pathway (NPP) in the erythrocyte membrane which allow the transport of nutrients and waste products necessary for the parasite (Kirk and Lehane 2014). Studies have shown that both parasite driven proteins encoded by *clag3*.*1* and *clag3.2* (Ekland, Akabas, and Fidock 2011; Nguitragool et al., 2011) and host benzodiazepine receptor mediate the formation of NPP (Bouyer, Egée, and Thomas 2006; Bouyer et al., 2011). Given that NPP can be a potential target for new therapeutics, further studies are needed to investigate the role of host variants in influencing the NPP channel formation and its ability transporting nutrients to the parasites.

One of the key virulence factors of *P*. *falciparum* is its capacity to modify iRBCs to adhere to the endothelium of the vasculature and, thereby, sequester in capillaries and postcapillary venules in vital organs leading to severe disease manifestations (Rowe et al., 2009). Adhesion phenotype is primarily mediated by expression of *P. falciparum* erythrocyte membrane protein 1 (*PfEMP1*) on the iRBCs (Heatwoie et al., 1995; Smith et al., 1995). The binding of iRBCs with endothelium involves various adhesion molecules including CD36, ICAM-1, E-selectin, and chondroitin sulfate A (CSA) that are variably expressed in different organs (Raventos-suarez et al., 1985; Fried and Duffy 1996; Rowe et al., 1997). The neural adhesion molecules identified in the current study might be involved in receptor activities, and their polymorphisms might play protective roles against SM. Adhesion events have been shown to activate Rho kinase signalling pathway which is strongly implicated in various vascular diseases (Taoufiq et al., 2008). The variants of genes that are enriched in these pathways in the current study might provide protection against severe malaria by weakening the cytoadherence interactions and associated pathologies. The variants of other genes that are enriched in neuronal system, axon guidance, and tight junction might be linked with intracerebral pathogenesis of SM (Nishanth and Schlüter 2019). Furthermore, the candidate malaria resistance genes identified by gene-based GWAS were differentially expressed in blood vessels, suggesting that the majority of the identified genes likely counteract *P*. *falciparum* induced endothelial disfunctions in microvasculature and capillaries (Moxon et al., 2020).

The PCA analysis effectively clustered the majority of the populations according to their ancestry. This suggests that the SNPs residing in the candidate genes are differentiated across geographical locations and ethnic background. However, the clusters generated from malaria endemic regions including Malawi and Gambia overlapped, likely because of the similar malaria endemicity in these populations. As expected, the MAF of common SNPs (MAF > 0.05) residing in the candidate genes is higher in the three malaria populations compared to the 20 global populations. However, it should be cautioned that the SNPs used here were not specifically associated with SM and may not represent causal and association SNP level frequency spectrum. Future association studies are needed to identify specific causal SNPs in these genes.

### Limitations

Severe malaria is a complex disease with various clinical manifestations including cerebral malaria, severe malarial anaemia, and others which may arise from distinct pathophysiological processes. This implies the existence of sub-phenotype specific variants that influence the disease outcomes. However, sub-phenotype analyses were not presented in the current study owing to the lack of adequate sub-phenotype information in the MalariaGen datasets. Moreover, our functional analysis, including Quantitative Trait Locus (eQTL) mapping, and chromatin interaction were based on datasets that come from European populations which might negatively affect the findings. Furthermore, given the high genetic diversity of African population, it could have been more appropriate to use population specific reference panel for each of the study population. Our genotype-based rare variant association analysis in the current study was limited because such analyses work best in whole genome dataset. Future whole genome-based studies are needed to better understand the contribution of rare variants in malaria resistance trait. Finally, our findings were not yet supported by experimental functional studies.

## Conclusion

Our functional mapping analysis identified 57 genes located in the known malaria genomic loci, while our gene-based GWAS analysis identified additional 125 genes across the genome which can potentially guide future experimental studies. The identified genes were significantly enriched in malaria pathogenic pathways including multiple overlapping pathways in erythrocyte-related functions, blood coagulations, ion channels, adhesion molecules, membrane signalling elements, and neuronal systems. Overall, our results suggest that severe malaria resistance trait is attributed to multiple genes that are enriched in overlapping pathways linked to severe malaria pathogenesis, highlighting the possibility of harnessing new malaria therapeutics that can simultaneously target multiple malaria protective host molecular pathways. Further experimental studies are needed to validate the findings in the current study.

## Data Availability

The original contributions presented in the study are included in the article/Supplementary Material, Further inquiries can be directed to the corresponding author.

## Supplementary Material

Supplementary Data 1 Describes the Genomic risk loci of severe malaria resistance, Independent significant SNPs and Lead SNPs identified from independent significant SNPs of severe malaria resistance GWAS, respectively. The genomic locus was defined as the region that contain independent lead SNPs and nominally significant SNPs (*p* < 0.05) in linkage dis-equilibrium (LD) block with lead SNPs. An independent significant SNP was defined as a genome-wide significant SNP (*P*-value < 5e^–8^) within the genomic boundary of LD threshold of r^2^ > 0.6.

Supplementary Data 2 Describes the Genomic risk loci of severe malaria resistance, Independent significant SNPs and Lead SNPs identified from independent significant SNPs of severe malaria resistance GWAS, respectively. The genomic locus was defined as the region that contain independent lead SNPs and nominally significant SNPs (p < 0.05) in linkage dis-equilibrium (LD) block with lead SNPs. An independent significant SNP was defined as a genome-wide significant SNP (P-value < 5e^–8^) within the genomic boundary of LD threshold of r^2^ > 0.6.

Supplementary Data 3 Describes the Genomic risk loci of severe malaria resistance, Independent significant SNPs and Lead SNPs identified from independent significant SNPs of severe malaria resistance GWAS, respectively. The genomic locus was defined as the region that contain independent lead SNPs and nominally significant SNPs (p < 0.05) in linkage dis-equilibrium (LD) block with lead SNPs. An independent significant SNP was defined as a genome-wide significant SNP (P-value < 5e^–8^) within the genomic boundary of LD threshold of r^2^ > 0.6.

Supplementary Data 4 Presents the description of prioritized genes from severe malaria resistance GWAS by three functional mapping strategies implemented in FUMA.

Supplementary Data 5 Describes malaria resistance candidate genes identified by gene-based GWAS analysis using Pascal method.

Supplementary Data 6 Indicates list of genes in which the proportion of pathogenic SNPs were much higher in the three malaria endemic populations (Kenya, Malawi and Gambia) compared to the global populations.

Supplementary Data 7 Presents the proportion of pathogenic SNPs in candidate malaria resistance genes for all the 23 global populations as identified by ANNOVAR.

Supplementary Figure 1 Chromatin interactions and eQTLs of severe malaria resistance candidate genes on chr 11 and chr 9 risk locus, respectively. The most outer layer is the Manhattan plot displaying SNPs with *P*-value < 0.05. Candidate SNPs are coloured based on the highest r^2^ to one of the independent significant loci (red: r^2^ > 0.8, orange: r^2^ > 0.6). Other SNPs are coloured in grey. The outer circle is the chromosome coordinate and genomic risk loci are highlighted in blue. Genes mapped by either Hi-C or eQTLs are shown on the inner circle. Genes identified by chromatin interaction and eQTLs are coloured orange and green respectively while genes mapped by both are coloured red.

Supplementary Figure 2 Chromatin interactions and eQTLs of severe malaria resistance candidate genes on chr 11 and chr 9 risk locus, respectively. The most outer layer is the Manhattan plot displaying SNPs with *P*-value < 0.05. Candidate SNPs are coloured based on the highest r^2^ to one of the independent significant loci (red: r^2^ > 0.8, orange: r^2^ > 0.6). Other SNPs are coloured in grey. The outer circle is the chromosome coordinate and genomic risk loci are highlighted in blue. Genes mapped by either Hi-C or eQTLs are shown on the inner circle. Genes identified by chromatin interaction and eQTLs are coloured orange and green respectively while genes mapped by both are coloured red.

Supplementary Figure 3 Shows MAF of SNPs for each candidate severe malaria resistance gene in three malaria endemic populations (Gambia, Malawi, Kenya) and global populations composed of 20 ethnic groups. Y-axis represent gene specific allele frequency, X-axis represent genes. Populations were represented by different colors and symbols.

Supplementary Figure 4 Shows MAF of SNPs for each candidate severe malaria resistance gene in three malaria endemic populations (Gambia, Malawi, Kenya) and global populations composed of 20 ethnic groups. Y-axis represent gene specific allele frequency, X-axis represent genes. Populations were represented by different colors and symbols.

SUPPLEMENTARY TABLE 1 Describes positional enrichment of genes identified by FUMA method using MsigDB genes as background.

SUPPLEMENTARY TABLE 2 Describes candidate malaria resistance genes identified by both functional mapping and gene-based GWAS analysis.

SUPPLEMENTARY TABLE 3 Describes candidate malaria resistance genes identified by both functional mapping and gene-based GWAS analysis.

## References

[B1] Achidi E. A. Agbenyega T. Allen S. Amodu O. Bojang K. Conway D. (2008). A Global Network for Investigating the Genomic Epidemiology of Malaria. Nature 456 (7223), 732–737. 10.1038/nature07632 19079050 PMC3758999

[B2] Adams Y. Kuhnrae P. Higgins M. K. Ghumra A. Rowe J. A. (2014). Rosetting Plasmodium Falciparum-Infected Erythrocytes Bind to Human Brain Microvascular Endothelial Cells *In Vitro*, Demonstrating a Dual Adhesion Phenotype Mediated by Distinct P. Falciparum Erythrocyte Membrane Protein 1 Domains. Infect. Immun. 82 (3), 949–959. 10.1128/IAI.01233-13 24343658 PMC3958005

[B3] Ahsan M. Weronica E. Rask-andersen M. Karlsson T. Lind-thomsen A. Enroth S. (2017). The Relative Contribution of DNA Methylation and Genetic Variants on Protein Biomarkers for Human Diseases. Plos Genet. 13 (9), e1007005. 10.1371/journal.pgen.1007005 28915241 PMC5617224

[B4] Alonso S. Lo S. Izagirre N. (2006). Overdominance in the Human Genome and Olfactory Receptor Activity. Mol. Biol. Evol. 25 (5), 997–1001. 10.1093/molbev/msn049 18296703

[B5] Andersson Robin. Gebhard C. Miguel-escalada I. Hoof I. Bornholdt J. Boyd M. (2014). An Atlas of Active Enhancers across Human Cell Types and Tissues. Nature 507, 455–461. 10.1038/nature12787 24670763 PMC5215096

[B6] Aprelikova O. Pajusola K. Partanen J. Armstrong E. Alitalo R. Bailey S. K. (1992). FLT4 a Novel Class III Receptor Tyrosine Kinase in Chromosome. Cancer Res. 52 (3), 746–748. 1310071

[B7] Astle W. J. Elding H. Jiang T. Ouwehand W. H. Butterworth A. S. Soranzo N. (2017). The Allelic Landscape of Human Blood Cell Trait Variation and Links to Common Complex Disease Resource the Allelic Landscape of Human Blood Cell Trait Variation and Links to Common Complex Disease. Cell 167 (5), 1415–1429. 10.1016/j.cell.2016.10.042 PMC5300907 27863252

[B8] Band G. Rockett K. A. Spencer C. A. Kwiatkowski D. P. Le Q. S. Clarke G. M. (2015). A Novel Locus of Resistance to Severe Malaria in a Region of Ancient Balancing Selection. Nature 526 (7572), 253–257. 10.1038/nature15390 26416757 PMC4629224

[B9] Bouyer G. Cueff A. Gallagher P. G. Thomas S. L. Y. (2011). Erythrocyte Peripheral Type Benzodiazepine Receptor/Voltage-dependent Anion Channels Are Upregulated by Plasmodium Falciparum. Blood 118 (8), 2305–2312. 10.1182/blood-2011-01-329300 21795748

[B10] Bouyer G. Egée S. Thomas S. L. (2006). Three Types of Spontaneously Active Anionic Channels in Malaria-Infected. Blood Cell Mol Dis 36 (2), 248–254. 10.1016/j.bcmd.2006.01.005 16510298

[B11] Boyle A. P. Hong E. L. Hariharan M. Cheng Y. Schaub M. A. Kasowski M. (2012). Annotation of Functional Variation in Personal Genomes Using RegulomeDB. Genome Res. 22 (9), 1790–1797. 10.1101/gr.137323.112.1790-97 22955989 PMC3431494

[B12] Brochet M. Billker O. (2016). Calcium Signalling in Malaria Parasites. Mol. Microbiol. 100 (3), 397–408. 10.1111/mmi.13324 26748879

[B13] Chen Kexin. Ma H. Li L. Zang R. Wang Ch. Song F. (2014). “Corrigendum: Genome-wide Association Study Identifies New Susceptibility Loci for Epithelial Ovarian Cancer in Han Chinese Women. Nat. Commun. 5, 5828. 10.1038/ncomms6828 25134534

[B14] Chimusa E. R. Mbiyavanga M. Mazandu G. K. Mulder N. J. (2015). AncGWAS: A Post Genome-wide Association Study Method for Interaction, Pathway and Ancestry Analysis in Homogeneous and Admixed Populations. Bioinformatics 32 (4), 549–556. 10.1093/bioinformatics/btv619 26508762 PMC5939890

[B15] Cowman A. F. Crabb B. S. (2006). Invasion of Red Blood Cells by Malaria Parasites. Cell 124, 755–766. 10.1016/j.cell.2006.02.006 16497586

[B16] Csardi G. Tamas N. (2006). The Igraph Software Package for Complex Network Research. Inter. J. Comp. Sys 1695, 1–9.

[B17] Damena D. Chimusa E. R. (2020). Genome-Wide Heritability Analysis of Severe Malaria Resistance Reveals Evidence of Polygenic Inheritance. Hum. Mol. Gen. 29 (1), 168–176. 10.1093/hmg/ddz258 31691794 PMC7416678

[B18] Damena D. Denis A. Golassa L. Chimusa E. R. (2019). “Genome-Wide Association Studies of Severe P . Falciparum Malaria Susceptibility : Progress , Pitfalls and Prospects. BMC Med. Genomics. 12 (120), 1–14. 10.1186/s12920-019-0564-x1-14 31409341 PMC6693204

[B19] Deelen P. Bonder M. J. Velde K. J. Westra H. Winder E. Hendriksen D. (2014). Genotype Harmonizer: Automatic Strand Alignment and Format Conversion for Genotype Data Integration. BMC. Res. Not. 7, 1–4. 10.1186/1756-0500-7-901 25495213 PMC4307387

[B20] Delaneau O. Marchini J. Zagury J. (2013). High-resolution Whole-Genome Haplotyping Using Limited Seed Data. Nat.Method. 9, 179–181. 10.1038/nmeth.2308 PMC3835542 23269372

[B21] Desai S. A. (2014). “Why Do Malaria Parasites Increase Host Erythrocyte Permeability. Trends Parasitol. 30 (3), 151–159. 10.1016/j.pt.2014.01.003 24507014 PMC3987781

[B22] Dudbridge F. (2016). Polygenic Epidemiology. Genet. Epidemiol. 40 (4), 268–272. 10.1002/gepi.21966 27061411 PMC4982028

[B23] Egan A. Keusch G. T. Breman J. G. (2001). The Intolerable burden of Malaria: a New Look at the Numbers. Am. J. Trop. Med. Hyg. 1824–1907. 10.4269/ajtmh.2001.64.iv 11425185

[B24] Ekland E. H. Akabas M. H. Fidock D. A. (2011). “Previews Taking Charge: Feeding Malaria via Anion Channels. Cell 145 (5), 645–647. 10.1016/j.cell.2011.05.012 21620131

[B25] Ernst J. Kellis M. (2012). ChromHMM: Automating Chromatin- State Discovery and Characterization. Nat. Methods 9, 215–216. 10.1038/nmeth.1906 22373907 PMC3577932

[B26] Fernandez T. Morgan T. Davis N. Klin A. Morris A. Farhi A. (2004). Disruption of Contactin 4 ( CNTN4 ) Results in Developmental Delay and Other Features of 3p Deletion Syndrome. Am. J. Hum. Genet. 74 (6), 1286–1293. 10.1086/421474.4:1286-93 15106122 PMC1182094

[B27] Foss S. Bottermann M. Jonsson A. Sandlie I. James L. C. Andersen J. T. (2019). TRIM21 — from Intracellular Immunity to Therapy. Front. Immuno 10, 2049. 10.3389/fimmu.2019.02049 PMC6722209 31555278

[B28] Francis S. E. Sullivan D. J. Goldberg D. E. (1997). Hemoglobin Metabolism in the Malaria Parasite *Plasmodium Falciparum* . Annu. Rev. Microbiol. 51, 97–123. 10.1146/annurev.micro.51.1.97.97-123 9343345

[B29] Francischetti I. Seydel K. B. Monteiro R. Q. (2008). Blood Coagulation , Inflammation , and Malaria. Microcirculation, 81–107. 10.1080/10739680701451516 18260002 PMC2892216

[B30] Fried M. Duffy P. E. (1996). Adherence of *Plasmodium Falciparum* to Chondroitin Sulfate A in the Human Placenta. Science 272 (5267), 1502–1504. 10.1126/science.272.5267.1502 8633247

[B31] Ghartey-kwansah G. Yin Q. Li Z. Gumpper K. Sun Y. Yang R. (2020). Calcium-dependent Protein Kinases in Malaria Parasite Development and Infection. Cell 29, 1–12. 10.1177/0963689719884888 PMC7444236 32180432

[B115] Giusti-Rodriguez P. M. Lu L. Yang Y. Crowley C. A. Liu X. Bryois J. (2018). Schizophrenia and a High-Resolution Map of the Three-Dimensional Chromatin Interactome of Adult and Fetal Cortex. bioRxiv, 406330.

[B32] Guerini D. Pan B. Carafoli E. (2003). Expression, Purification, and Characterization of Isoform 1 of the Plasma Membrane Ca2+ Pump. Focus on Calpain Sensitivity. J. Biolog Chem. 278 (40), 38141–38148. 10.1074/jbc.M302400200 12851406

[B33] Gulden F. O. Pochareddy S. Sunkin S. M. Li Zh. Shin Y. Pletikos M. (2018). Integrative Functional Genomic Analysis of Human Brain Development and Neuropsychiatric Risks. Science 362 (1264), 1–13. 10.1126/science.aat7615 PMC6413317 30545854

[B34] Gurdasani D. Carstensen T. Tekola-Ayele F. Pagani L. Tachmazidou I. Hatzikotoulas K. (2015). The African Genome Variation Project Shapes Medical Genetics in Africa. Nature 517 (7534), 327–332. 10.1038/nature13997 25470054 PMC4297536

[B35] Hagberg A. A., and Los Alamos National Laboratory (2008). Exploring Network Structure, Dynamics, and Function Using Network. XSciPy 7, 11–15. Available at: http://conference.scipy.org/proceedings/SciPy2008/paper_2 .

[B36] Hamada N. Ito H. Nishijo T. Iwamoto I. Morishita R. (2016). Essential Role of the Nuclear Isoform of RBFOX1, a Candidate Gene for Autism Spectrum Disordersthe Brain Development. Sci. Rep. 6, 1–19. 10.1038/srep30805 27481563 PMC4969621

[B37] Heatwoie V. M. Wertheimer S. P. Peterson D. S. Ravetch J. A. Weilems T. E. (1995). The Large Diverse Gene Family W Encodes Proteins Involved in Cytoadherence and Antigenic Variation of Plasmodium Falciparum-Infected Erythrocytes. Cell 82 (1), 89–100. 7606788 10.1016/0092-8674(95)90055-1

[B38] Hedrick P. (2011). Population Genetics of Malaria Resistance in Humans. Heredity 10716, 283–304. 10.1038/hdy.2011.16 PMC3182497 21427751

[B39] Hellberg A. Poole J. Olsson M. L. (2002). Molecular Basis of the Globoside-Deficient Pk Blood Group. J. Biol. Chem. 277 (33), 29455–29459. 10.1074/jbc.M203047200 12023287

[B40] Hoffman M. Ernst J. Wilder S. P. Kundaje A. Harris R. S. Libbrecht M. (2013). Integrative Annotation of Chromatin Elements from ENCODE Data. Nucl. Acids Res. 41 (2), 827–841. 10.1093/nar/gks1284 23221638 PMC3553955

[B41] Hollestelle M. J. Mantey E. A. Chakravorty S. J. Craig A. Akoto A. O. Donnell J. O. (2006). Von Willebrand Factor Propeptide in Malaria: Evidence of Acute Endothelial Cell Activation. Br. J. Haemat 133, 562–569. 10.1111/j.1365-2141.2006.06067.x 16681646

[B42] Howie B. N. Donnelly P. Marchini J. (2009). A Flexible and Accurate Genotype Imputation Method for the Next Generation of Genome-wide Association Studies. Plos Genet. 5, e1000529. 10.1371/journal.pgen.1000529 19543373 PMC2689936

[B43] Huckins L. M. Dobbyn A. Ruderfer D. M. Hoffman G. Wang W. Pardiñas A. F. (2019). Regions Provides Insights into Schizophrenia Risk. Nat. Genet. 51, 659–674. 10.1038/s41588-019-0364-4 30911161 PMC7034316

[B44] Hunt N. H. Ball H. J. Hansen A. M. Khaw L. T. Guo J. Bakmiwewa S. (2014). “Cerebral Malaria : Gamma-Interferon Redux. Front. Cellul Infec Microbiol. 4, 1–12. 10.3389/fcimb.2014.00113 PMC4133756 25177551

[B45] Ionita-laza I. Lee S. Makarov V. Buxbaum J. D. Lin X. (2013). Sequence Kernel Association Tests for the Combined Effect of Rare and Common Variants. Am. J. Hum. Genet. Am. Soc. Hum. Genet. 92, 841–853. 10.1016/j.ajhg.2013.04.015 PMC3675243 23684009

[B46] Jallow M. Teo Y. Small K. S. Rockett K. A. Clark T. G. Kivinen K. (2010). Genome-Wide and Fine-Resolution Association Analysis of Malaria in West Africa. Nat. Genet 41 (6), 657–665. 10.1038/ng.388.Genome-wide PMC2889040 19465909

[B47] Jiao X. Sherman B. T. Huang D. W. Stephens R. Baseler M. W. Lane H. C. (2012). “DAVID-WS : A Stateful Web Service to Facilitate Gene/Protein List Analysis”. Bioinformatics 28 (13), 1805–1806. 10.1093/bioinformatics/bts251 22543366 PMC3381967

[B48] Jonscher E. Flemming S. Schmitt M. Spielmann T. Jonscher E. Flemming S. (2019). PfVPS45 Is Required for Host Cell Cytosol Uptake by Malaria Blood Stage Parasites. Cell Host Microb 25 (3), 166–173. 10.1016/j.chom.2018.11.010 30581113

[B49] Kevin E. B. Jonathan R. H. Giorgio G. Stancie M. A. Elton D. L. Peggy M. (1994). Resistance to Parvovirus B19 Infection Due to Lack of Virus Receptor Erythrocyte Antigen. N. Engl. J. Med. 330, 1192–1196. 8139629 10.1056/NEJM199404283301704

[B50] Kevin M. Dayo F. Catherine W. Isiah M. Maria W. Victoria M. (1995). Indicators of Life-Threatening Malaria in African Children. N. Engl. J. Med. 332 (21), 1399–1404. 10.1056/NEJM199505253322102 7723795

[B52] Kircher M. Witten D. M. Jain P. Roak B. J. O. Cooper G. M. Shendure J. (2014). A General Framework for Estimating the Relative Pathogenicity of Human Genetic Variants. Nat. Genet. 46, 310–315. 10.1038/ng.2892 24487276 PMC3992975

[B53] Kirk K. Lehane A. M. (2014). Membrane Transport in the Malaria Parasite and its Host Erythrocyte. Biochem. J. 457 18 (1), 1–18. 10.1042/BJ20131007 24325549

[B54] Kraisin S. Naka I. Patarapotikul J. Nantakomol D. Nuchnoi P. (2011). Association of ADAMTS13 Polymorphism with Cerebral Malaria. Mal J. 10, 1–8. 10.1186/1475-2875-10-366 PMC3261218 22168261

[B55] Kristiansen M. Graversen J. H. Jacobsen Ch. Hoffman E. Law S. K. A. Sonne O. (2001). Identification of the Haemoglobin Scavenger Receptor. Nature 409, 198–201. 10.1038/35051594 11196644

[B56] Kutmon M. Riutta A. Nunes N. Hanspers K. Willighagen E. L. Bohler A. (2016). WikiPathways: Capturing the Full Diversity of Pathway Knowledge. Nucl. Acids Res. 44, 488–494. 10.1093/nar/gkv1024 PMC4702772 26481357

[B57] Kwiatkowski D. P. (2005). How Malaria Has Affected the Human Genome and what Human Genetics Can Teach Us about Malaria. Am. J. Hum. Genet. 77 (2), 171–192. 10.1086/432519 16001361 PMC1224522

[B58] Lamparter D. Marbach D. Rueedi R. Kutalik Z. (2016). Fast and Rigorous Computation of Gene and Pathway Scores from SNP-Based Summary Statistics. Plos Genet. 12 (1), e1004714. 10.1371/journal.pcbi.1004714 PMC4726509 26808494

[B59] Lee A. S. Rusch J. Lima A. C. Usmani A. Huang N. Lepamets M. (2019). Rare Mutations in the Complement Regulatory Gene CSMD1 Are Associated with Male and Female Infertility. Nat. Commun. 10, 1–16. 10.1038/s41467-019-12522-w 31604923 PMC6789153

[B60] Leeuw Ch. Mooij J. M. Heskes T. Posthuma D. (2015). MAGMA: Generalized Gene-Set Analysis of GWAS Data. Plos Genet. 11, e1004219. 10.1371/journal.pcbi.1004219 PMC4401657 25885710

[B61] Liberzon A. Subramanian A. Pinchback R. Thorvaldsdóttir H. Tamayo P. Mesirov J. P. (2011). Molecular Signatures Database ( MSigDB ) 3 . 0. Bioinformatics 27 (12), 1739–1740. 10.1093/bioinformatics/btr260 21546393 PMC3106198

[B62] Lund B. Lindberg F. P. Baga M. Normark S. (1985). Globoside-Specific Adhesins of Uropathogenic Escherichia Coli Are Encoded by Similar Trans-complementable Gene Clusters. J. Bacteriol. 162 (3), 1293–1301. 10.1128/jb.162.3.1293-1301.1985 2860097 PMC215918

[B63] Malaria Genomic Epidemiology Network (2019). Insights into Malaria Susceptibility Using Genome-wide Data on 17,000 Individuals from Africa, Asia and Oceania. Nat. Commun. 10, 5732. 10.1038/s41467-019-13480-z 31844061 PMC6914791

[B64] Marees A. T. Stringer S. Claire M. Derks E. M. (2018). “A Tutorial on Conducting Genome-wide Association Studies : Quality Control and Statistical Analysis. Int. J. Methods Psychiatr. Res. 27 (2), 1–10. 10.1002/mpr.1608 PMC6001694 29484742

[B65] Mbengue A. Yam X. Y. Braun-breton C. (2012). Human Erythrocyte Remodelling during Plasmodium Falciparum Malaria Parasite Growth and Egress,. Br. J. Haematol. 157 (2), 171–179. 10.1111/j.1365-2141.2012.09044.x 22313394

[B66] Mccarthy E. M. Joan N. Kallal L. E. Wynne C. Lazzari E. Higgs R. (2014). TRIM68 Negatively Regulates IFN- B Production by Degrading TRK Fused Gene , a Novel Driver of IFN- B Downstream of Anti-viral Detection Systems. PloS ONE 9 (7), e101503. 10.1371/journal.pone.0101503 24999993 PMC4084880

[B67] Milet J. Boland A. Luisi P. Sabbagh A. Sadissou I. Sonon P. (2019). First Genome-wide Association Study of Non-severe Malaria in Two Birth Cohorts in Benin. Hum. Genet. 138 (11-12), 1341–1357. 10.1007/s00439-019-02079-5 31667592

[B68] Miller L. H. Baruch D. I. Marsh K. Doumbo O. K. (2002). The Pathogenic Basis of Malaria. Nature 415 (6872), 673–679. 10.1038/415673a 11832955

[B69] Millholland M. G. Satish M. Dupont C. D. Love M. S. Patel B. Shilling D. (2013). A Host GPCR Signaling Network Required for the Cytolysis of Infected Cells Facilitates Release of Apicomplexan Parasites. Cel Host Microb 13 (1), 15–28. 10.1016/j.chom.2012.12.001 PMC3638031 23332153

[B70] Milner D. A. (2018). Malaria Pathogenesis,. Cold Spr Harb Perspect. Med. 8 (1), a025569. 10.1101/cshperspect.a025569 PMC5749143 28533315

[B71] Mostafavi S. Ray D. Warde-farley D. Grouios Ch. Morris Q. (2008). GeneMANIA: A Real-Time Multiple Association Network Integration Algorithm for Predicting Gene Function. Genome Biol. 9, 1–15. 10.1186/gb-2008-9-s1-s4 PMC2447538 18613948

[B72] Moxon A. Matthew P. G. McGuinness D. Danny A. M. Matthias M. (2020). New Insights into Malaria Pathogenesis. Annu. Rev. Pathol. Mech. Dis. 15, 315–343. 10.1146/annurev-pathmechdis-012419-032640 31648610

[B73] Mrozek K. Tanner S. M. Heinonen K. Bloomfield C. D. (2003). Molecular Cytogenetic Characterization of the KG-1 and KG-1a Acute Myeloid Leukemia Cell Lines by Use of Spectral Karyotyping and Fluorescence *In Situ* Hybridization. Genes Chrom Canc 252, 249–252. 10.1002/gcc.10274 14506699

[B74] Nguitragool W. Bokhari A. Pillai A. D. Rayavara K. Sharma P. Turpin B. (2011). Malaria Parasite Clag3 Genes Determine Channel-Mediated Nutrient Uptake by Infected Red Blood Cells. Cell 145 (5), 665–677. 10.1016/j.cell.2011.05.002 21620134 PMC3105333

[B75] Nishanth G. Schlüter D. (2019). “Blood-Brain Barrier in Cerebral Malaria: Pathogenesis and Therapeutic Intervention. Trends Parasitol. 35 (7), 516–528. 10.1016/j.pt.2019.04.010 31147271

[B76] Parker M. Bull S. J. Vries J. D. Agbenyega T. Doumbo O. K. Kwiatkowski D. P. (2009). Ethical Data Release in Genome-wide Association Studies in Developing Countries. Plos Med. 6 (11), 4–7. 10.1371/journal.pmed.1000143 PMC2771895 19956792

[B77] Patterson N. Price A. L. Reich D. (2006). Population Structure and Eigenanalysis. Plos Genet. 2 (12), e190. 10.1371/journal.pgen.0020190 17194218 PMC1713260

[B78] Pillai A. D. Addo R. Sharma P. Nguitragool W. Srinivasan P. Desai S. A. (2013). Malaria Parasites Tolerate a Broad Range of Ionic Environments and Do Not Require Host Cation Remodelling. Mol. Microbiol. 88 (1), 20–34. 10.1111/mmi.12159 23347042 PMC3608822

[B79] Purcell S. Neale B. Todd-Brown K. Thomas L. Ferreira M. A. R. Bender D. (2007). PLINK: A Tool Set for Whole-Genome Association and Population-Based Linkage Analyses. Am. J. Hum. Genet. 81 (3), 559–575. 10.1086/519795 17701901 PMC1950838

[B80] Ramasamy A. Trabzuni D. Guelfi S. Varghese V. Smith C. Walker R. (2014). Genetic Variability in the Regulation of Gene Expression in Ten Regions of the Human Brain. Nat. Neuro Sci. 17 (10), 1418–1428. 10.1038/nn.3801 PMC4208299 25174004

[B81] Ravenhall M. Campino S. Sepu N. Nadjm B. Mtove G. Wangai H. (2018). Novel Genetic Polymorphisms Associated with Severe Malaria and under Selective Pressure in North-Eastern Tanzania. Plos Genet. 14 (1), e1007172. 10.1371/journal.pgen.1007172 29381699 PMC5806895

[B82] Raventos-suarez C. Kaul D. K. Macalusot F. Nagel R. L. (1985). Membrane Knobs Are Required for the Microcirculatory Obstruction Induced by Plasmodium Falciparum-Infected Erythrocytes. Proc. Nadl. Acad. Sci. 82, 3829–3833. 10.1073/pnas.82.11.3829 PMC397881 3889917

[B83] Roadmap Epigenomics Consortium (2015). Integrative Analysis of 111 Reference Human Epigenomes. Nature 48, 317–330. 10.1038/nature1424810.1038/nature14248 PMC4530010 25693563

[B84] Rowe J. A. Claessens A. Corrigan R. A. Arman M. (2009). Adhesion of Plasmodium Falciparum-Infected Erythrocytes to Human Cells: Molecular Mechanisms and Therapeutic Implications. Exp. Rev. Mol. Med. 11, e16. 10.1017/s1462399409001082 PMC2878476 19467172

[B85] Rowe J. A. Moulds J. M. Newbold C. Miller L. H. (1997). Mediated by a Parasite-Variant Erythrocyte Membrane Protein and Complement-Receptor 1. Nature 388, 292–295. 10.1038/40888 9230440

[B86] Sabater-Lleal M. Jennifer E. H. Paul Sd S. Jonathan M. (2019). Genome-Wide Association Transethnic Meta-Analyses Identifies Novel Associations Regulating Coagulation Factor VIII and von Willebrand Factor Plasma Levels. Circulation 139 (5), 620–635. 10.1161/circulationaha.118.034532 30586737 PMC6438386

[B87] Schmiedel B. Singh D. Madrigal A. Kronenberg M. Schmiedel B. J. Singh D. (2018). Impact of Genetic Polymorphisms on Human Immune Resource Impact of Genetic Polymorphisms on Human Immune Cell Gene Expression. Cell 175 (6), 1701–1715. 10.1016/j.cell.2018.10.022 30449622 PMC6289654

[B88] Schmitt Anthony. D. Hu Ming. Jung Inkyung. Lin Yiing. Barr Cathy. L. (2016). A Compendium of Chromatin Contact Maps Reveals Spatially Active Regions in the Human Genome. Cell Rep. 17 (8), 2042–2059. 10.1016/j.celrep.2016.10.061 27851967 PMC5478386

[B89] Sicard A. Semblat J. Doerig C. Hamelin R. Moniatte M. Dorin-semblat D. (2011). Activation of a PAK-MEK Signalling Pathway in Malaria Parasite-Infected Erythrocytes. Cell Microbiol 13, 836–845. 10.1111/j.1462-5822.2011.01582.x 21371233 PMC3123749

[B90] Smith J. D. Craig A. G. Roberts D. J. Hudson-taylor D. E. Peterson D. S. Pinches R. (1995). Switches in Expression of Plasmodium Falciparum VW Genes Correlate with Changes in Antigenic and Cytoadherent Phenotypes of Infected Erythrocytes. Cell 82, 101–110. 10.1016/0092-8674(95)90056-x 7606775 PMC3730239

[B91] Sporn L. A. Victor J. M. Wagner D. Stel H. V. Mourik J. A. V. (1989). Differing Polarity of the Constitutive and Regulated Secretory Pathways for von Willebrand Factor in Endothelial Cells. J. Cel Biol 108 (4), 1283–1289. 10.1083/jcb.108.4.1283 PMC2115502 2494192

[B92] Sullivan J. M. O. Preston R. J. S. Regan N. O. O Donnell J. S. (2016). Emerging Roles for Hemostatic Dysfunction in Malaria Pathogenesis. Blood 127 (19), 2281–2289. 10.1182/blood-2015-11-636464 26851291

[B93] Sun P. C. Uppaluri R. Schmidt A. P. Pashia M. E. Quant E. C. Sunwoo J. B. (2001). Transcript Map of the 8p23 Putative Tumor Suppressor Region. Genomics 75, 17–25. 10.1006/geno.2001.6587 11472063

[B94] Taoufiq Z. Gay F. Balvanyos J. Ciceron L. Tefit M. Lechat P. (2008). “Rho Kinase Inhibition in Severe Malaria : Thwarting Parasite-Induced Collateral Damage to Endothelia”. J. Infect. Dis. 197 (7), 1062–1073. 10.1086/528988 18419473

[B95] Taylor S. M. Cerami C. Fairhurst R. M. (2013). Hemoglobinopathies: Slicing the Gordian Knot of Plasmodium Falciparum Malaria Pathogenesis. PLoS Pathog. 9 (5). 10.1371/journal.ppat.1003327 PMC3656091 23696730

[B96] Teo Y. Small K. Kwiatkowski D. P. (2013). Methodological Challenges of Genome-wide Association Analysis in Africa. Nat. Rev. Genet. 11 (2), 149–160. 10.1038/nrg2731.Methodological PMC3769612 20084087

[B97] The 1000 Genomes Project Consortium (2011). A Map of Human Genome Variation from Population Scale Sequencing. Nature 467 (7319), 1061–1073. 10.1038/nature09534.A PMC3042601 20981092

[B99] The GTex Consortium (2015). The Genotype-Tissue Expression (GTEx) Pilot Analysis 2015: Multitissue Gene Regulation in Humans. Science 348 (6235), 648–661. 10.1126/science.1262110 25954001 PMC4547484

[B100] Tiffert T. Lew V. L. Ginsburg H. Krugliak M. Croisille L. Mohandas N. (2005). The Hydration State of Human Red Blood Cells and Their Susceptibility to Invasion by Plasmodium Falciparum. Blood 105 (12), 4853–4860. 10.1182/blood-2004-12-4948 15728121 PMC1894996

[B101] Timmann C. Thye T. Vens M. Evans J. May J. Ehmen C. (2012). Genome-Wide Association Study Indicates Two Novel Resistance Loci for Severe Malaria. Nature 489 (7416), 443–446. 10.1038/nature11334 22895189

[B102] Tosevski V. Forman R. Muller W. Couper N. (2017). Crossm Gamma Interferon Mediates Experimental Cerebral Malaria by Signaling within Both the Hematopoietic and Nonhematopoietic Compartments. Am. Soc. Microbiol. 85 (11), 1–13. 10.1128/IAI.01035-16 PMC5649021 28874445

[B103] Trampuz A. Jereb M. Muzlovic I. Prabhu R. M. (2003). Clinical Review: Severe Malaria. Crit. Care 7 (4), 315–323. 10.1186/cc2183 12930555 PMC270697

[B104] Võsa U. Claringbould A. Westra H. Bonder M. J. Zeng B. Kirsten H. (2018). Unraveling the Polygenic Architecture of Complex Traits Using Blood EQTL Meta- Analysis. bioRxiv, 1–57. 10.1101/447367

[B105] Wang K. Li M. Hakonarson H. (2010). ANNOVAR: Functional Annotation of Genetic Variants From High-Throughput Sequencing Data. Nucl. Acids Res. 38 (16), 1–7. 10.1093/nar/gkq603 20601685 PMC2938201

[B106] Watanabe K. Taskesen E. Bochoven A. V. Posthuma D. (2017). Functional Mapping and Annotation of Genetic Associations with FUMA. Nat. Commun. 8, 1826. 10.1038/s41467-017-01261-5 29184056 PMC5705698

[B107] Wijst M. G. P. Brugge H. de Vries D. H. Deelen P. Swertz M. A. (2018). Single-cell RNA Sequencing Identifies Celltype-specific Cis-eQTLs and Co-expression QTLs. Nat. Genet. 50, 493–497. 10.1038/s41588-018-0089-9 29610479 PMC5905669

[B108] World Health Organization (2014). Severe Malaria. Trop. Med. Int. Healt 19, 7–131. 10.1111/tmi.12313 25214480

[B109] World Health Organization (2018). World Malaria Report 2018

[B110] World Health Organization (2019). World Malaria Report 2019

[B111] Yeo T. W. Lampah D. A. Gitawati R. Tjitra E. Kenangalem E. Piera K. (2008). Angiopoietin-2 Is Associated with Decreased Endothelial Nitric Oxide and Poor Clinical Outcome in Severe Falciparum Malaria. PNAS 105 (44), 17097–17102. 10.1073/pnas.0805782105 18957536 PMC2575222

[B112] Yoon S. Nguyen H. C. T. Yoo Y. J. Kim J. Baik B. Kim S. (2018). Efficient Pathway Enrichment and Network Analysis of GWAS Summary Data Using GSA-SNP2. Nucl. Acids Res. 46 (10), e60. 10.1093/nar/gky175 29562348 PMC6007455

[B113] Zheng X. L. (2016). ADAMTS13 and von Willebrand Factor in Thrombotic Thrombocytopenic Purpura. Annu. Rev. Med. 66, 211–225. 10.1146/annurev-med-061813-01324110.1146/annurev-med-061813-013241 PMC4599565 25587650

[B114] Zhernakova D. V. Deelen P. Vermaat M. Iterson M. V. Galen M. V. Arindrarto W. (2016). Identification of Context-dependent Expression Quantitative Trait Loci in Whole Blood. Nat. Genet. 49, 139–145. 10.1038/ng.3737 27918533

