# Supplementary material for: Insilico Functional Analysis of Genome-Wide Dataset From 17,000 Individuals Identifies Candidate Malaria Resistance Genes Enriched in Malaria Pathogenic Pathways
Source: Front Genet. 2021 Nov 18;12:676960. doi: 10.3389/fgene.2021.676960 (PMC8639191; doi:10.3389/fgene.2021.676960)
Supplement: Supplementary file 3 [file Table12.DOCX]

1. **Description of supplementary information**
   1. **Description of supplementary data**

**Supplementary Data 1-3** Describes the Genomic risk loci of severe malaria resistance, Independent significant SNPs and Lead SNPs identified from independent significant SNPs of severe malaria resistance GWAS, respectively. The genomic locus was defined as the region that contain independent lead SNPs and nominally significant SNPs (p<0.05) in linkage dis-equilibrium (LD) block with lead SNPs. An independent significant SNP was defined as a genome-wide significant SNP (P-value < 5e-8) within the genomic boundary of LD threshold of r2 > 0.6.

**Supplementary Data 4** presents the description of prioritized genes from severe malaria resistance GWAS by three functional mapping strategies implemented in FUMA. While **Supplementary Data 5** describes malaria resistance candidate genes identified by gene-based GWAS analysis using Pascal method.

**Supplementary Data 6** indicates list of genes in which the proportion of pathogenic SNPs were much higher in the three malaria endemic populations (Kenya, Malawi and Gambia) compared to the global populations while Supplementary **Data 7** presents the proportion of pathogenic SNPs in candidate malaria resistance genes for all the 23 global populations as identified by ANNOVAR.

- 1. **Description of supplementary Figures**

**Supplementary Figure 1 and 2**. Chromatin interactions and eQTLs of severe malaria resistance candidate genes on chr 11 and chr 9 risk locus, respectively. The most outer layer is the Manhattan plot displaying SNPs with P-value < 0.05. Candidate SNPs are coloured based on the highest r2 to one of the independent significant loci (red: r2 > 0.8, orange: r2 > 0.6). Other SNPs are coloured in grey. The outer circle is the chromosome coordinate and genomic risk loci are highlighted in blue. Genes mapped by either Hi-C or eQTLs are shown on the inner circle. Genes identified by chromatin interaction and eQTLs are coloured orange and green respectively while genes mapped by both are coloured red.

**Supplementary Figure 3**

Shows MAF of SNPs for each candidate severe malaria resistance gene in three malaria endemic populations (Gambia, Malawi, Kenya) and global populations composed of 20 ethnic groups. Y-axis represent gene specific allele frequency, X-axis represent genes. Populations were represented by different colors and symbols.

- 1. **Description of supplementary Tables**

**Supplementary Table 1**. Describes positional enrichment of genes identified by FUMA method using MsigDB genes as background. **Supplementary Table 2** describes candidate malaria resistance genes identified by both functional mapping and gene-based GWAS analysis. **Supplementary Table 3.** Describes data obtained from 1000 Genomes Project (1KGP) (Consortium et al.,2012) and the African Genome Variation Project (AGVP) (Gurdasani et al.,2015) and used for analysis.
