# Supplementary material for: Insilico Functional Analysis of Genome-Wide Dataset From 17,000 Individuals Identifies Candidate Malaria Resistance Genes Enriched in Malaria Pathogenic Pathways
Source: Front Genet. 2021 Nov 18;12:676960. doi: 10.3389/fgene.2021.676960 (PMC8639191; doi:10.3389/fgene.2021.676960)
Supplement: Supplementary file 4 [file Table9.DOCX]

**S 1**. Positional enrichment of candidate malaria resistance genes identified by FUMA method using MsigDB genes as background

| **Position** | **N** | **N** | **P-value** | **adjusted P** | **Genes** |
| --- | --- | --- | --- | --- | --- |
| [chr11p15](http://www.gsea-msigdb.org/gsea/msigdb/cards/chr11p15) | 297 | 19 | 8.86e-21 | 2.65e-18 | *TRIM21, TRIM68, MMP26, OR51F2, OR51L1, OR52A1, HBB, HBD, HBG1, HBG2, HBE1, OR51B2, UBQLNL, TRIM6, TRIM6-TRIM34, TRIM34, TRIM22, CCKBR, PRKCDBP* |
| [chr9q34](http://www.gsea-msigdb.org/gsea/msigdb/cards/chr9q34) | 214 | 11 | 3.10e-11 | 4.63e-9 | *RALGDS, GBGT1, SURF6, MED22, SURF1, SURF2, SURF4, REXO4, ADAMTS13, SLC2A6, ADAMTSL2* |
| [chr4q31](http://www.gsea-msigdb.org/gsea/msigdb/cards/chr4q31) | 67 | 6 | 4.61e-8 | 4.60e-6 | *RNF150, ZNF330, GAB1, FREM3, GYPE, GYPB* |
| [chr1q32](http://www.gsea-msigdb.org/gsea/msigdb/cards/chr1q32) | 147 | 7 | 2.62e-7 | 1.96e-5 | *CHIT1, BTG2, FMOD, PRELP, ATP2B4, LAX1, PLEKHA6* |
| [chr3q26](http://www.gsea-msigdb.org/gsea/msigdb/cards/chr3q26) | 61 | 3 | 7.97e-4 | 4.77e-2 | *B3GALNT1, NMD3, SPTSSB* |
