# Supplementary figures and images for: Insilico Functional Analysis of Genome-Wide Dataset From 17,000 Individuals Identifies Candidate Malaria Resistance Genes Enriched in Malaria Pathogenic Pathways
Source: Front Genet. 2021 Nov 18;12:676960. doi: 10.3389/fgene.2021.676960 (PMC8639191; doi:10.3389/fgene.2021.676960)

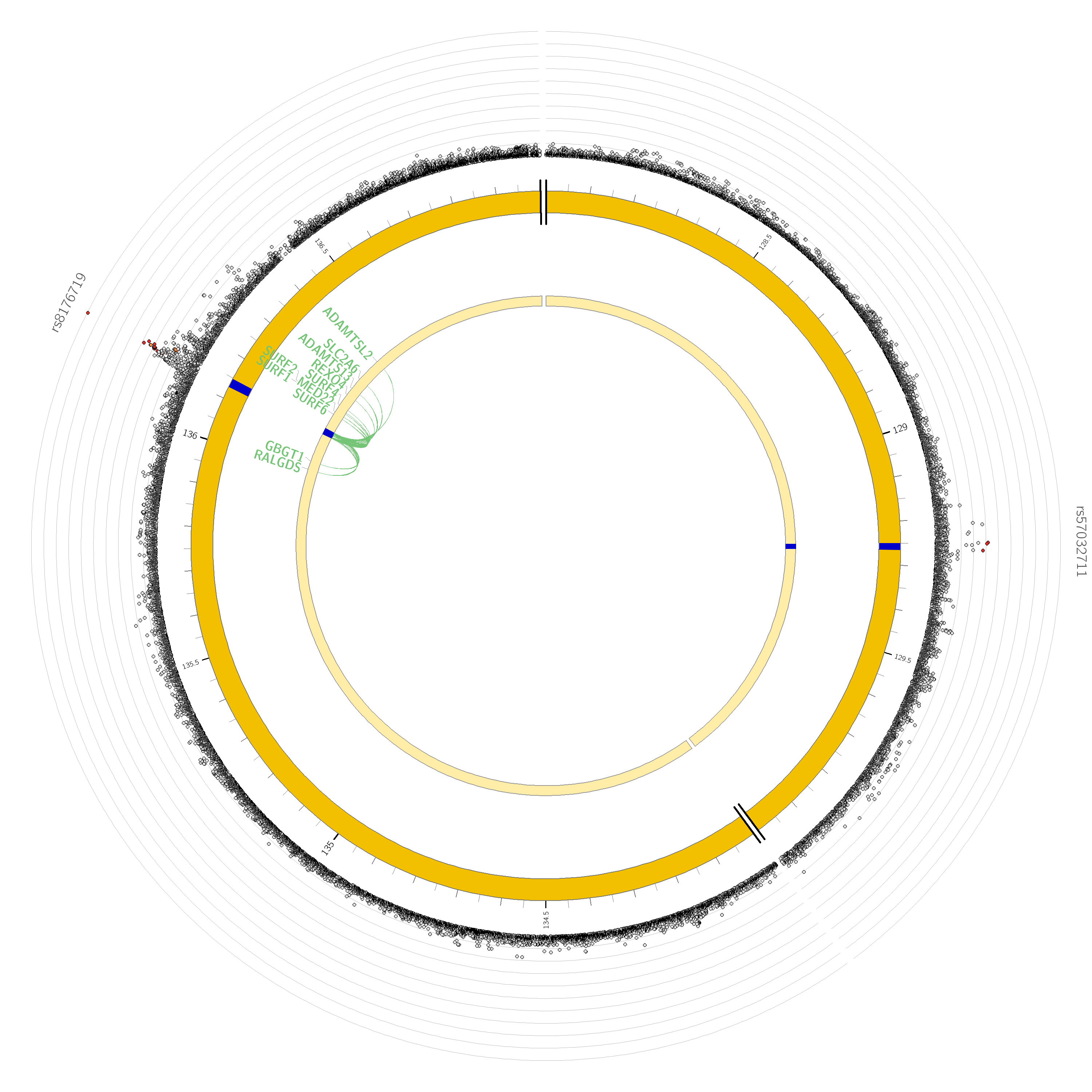

Supplement: Supplementary file 7 [file Image2.PNG]

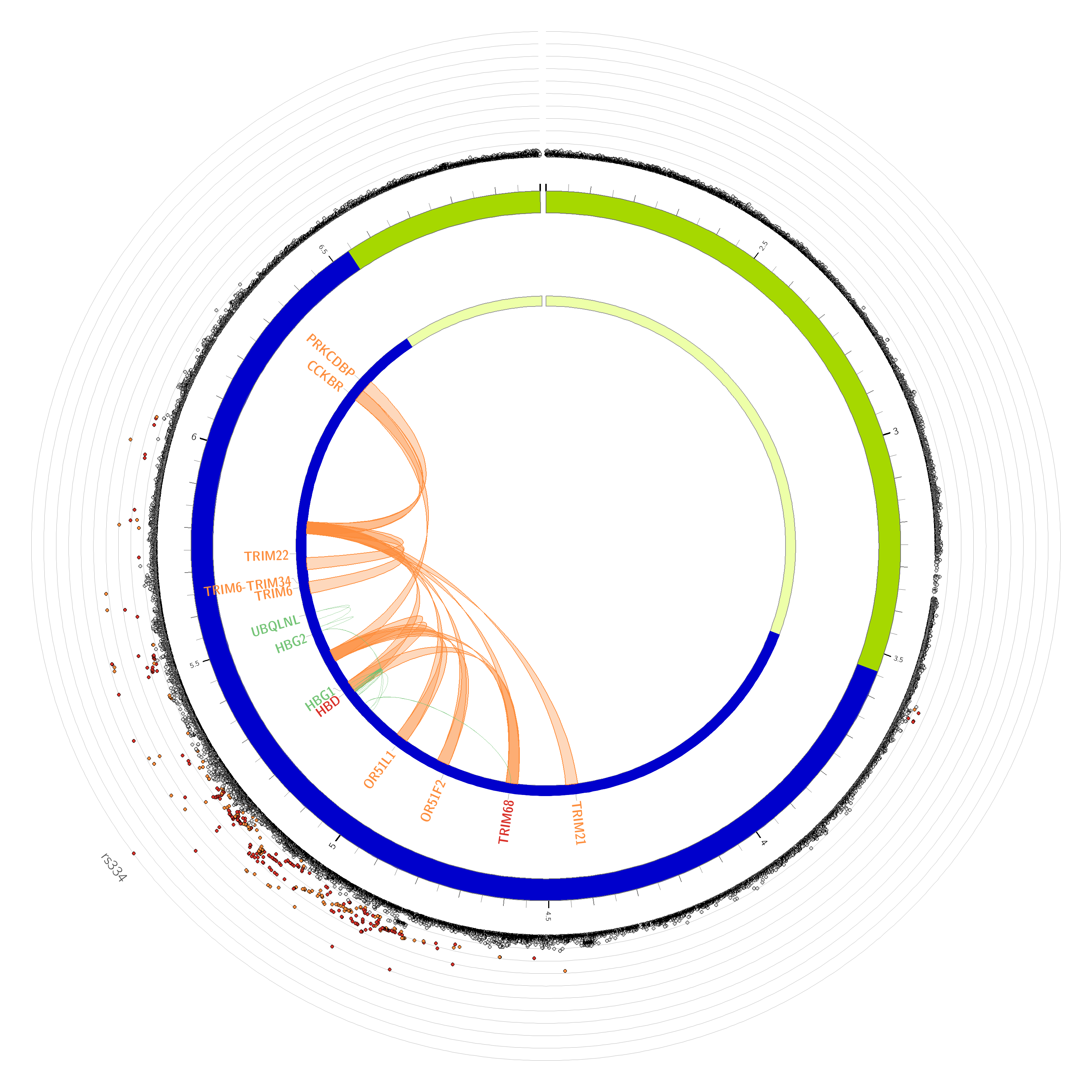

Supplement: Supplementary file 9 [file Image1.PNG]

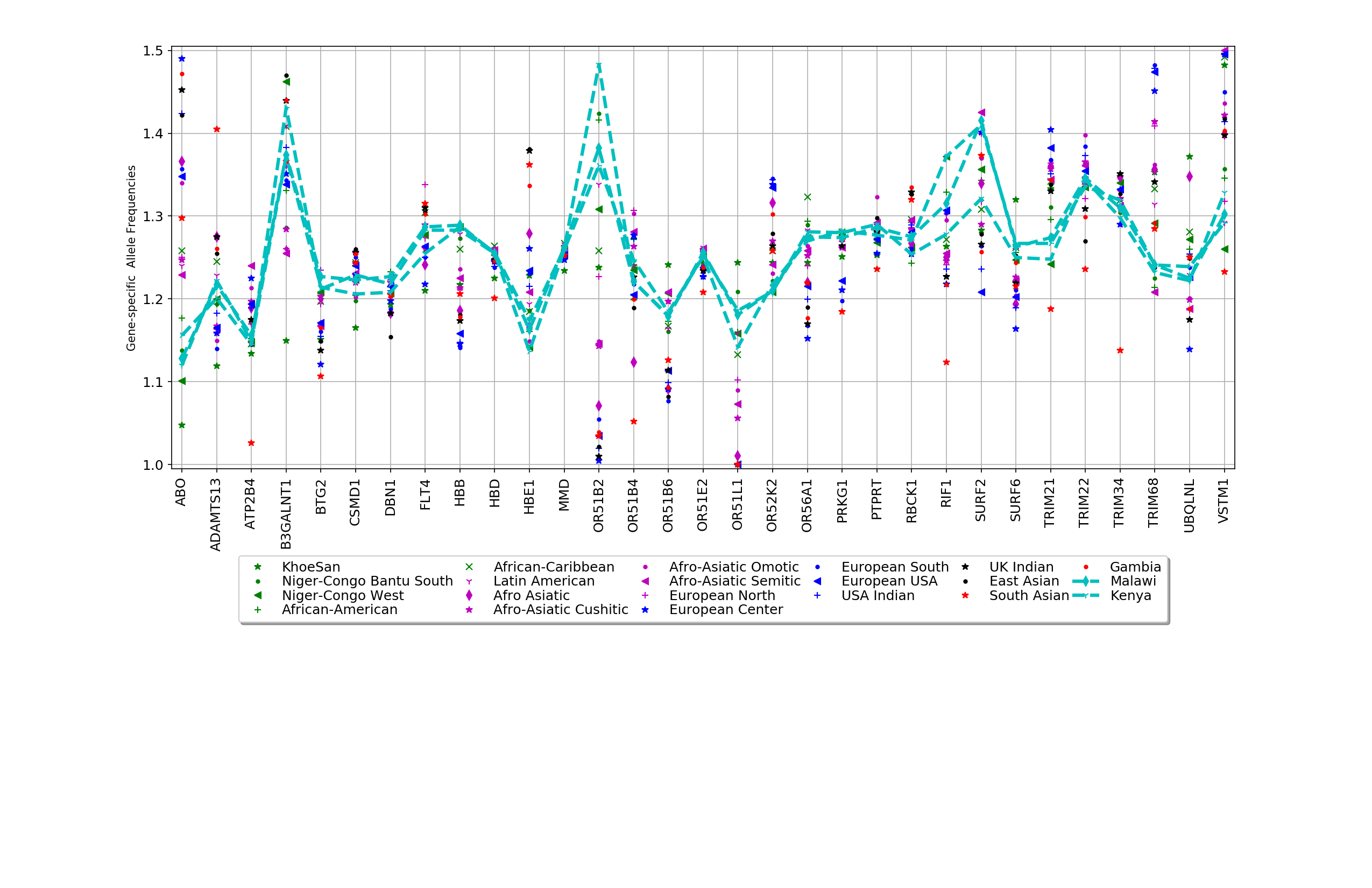

Supplement: Supplementary file 14 [file Image3.PNG]
