# Supplementary material for: Insilico Functional Analysis of Genome-Wide Dataset From 17,000 Individuals Identifies Candidate Malaria Resistance Genes Enriched in Malaria Pathogenic Pathways
Source: Front Genet. 2021 Nov 18;12:676960. doi: 10.3389/fgene.2021.676960 (PMC8639191; doi:10.3389/fgene.2021.676960)
Supplement: Supplementary file 10 [file Table11.DOCX]

S3 Table. Data obtained from 1000 Genomes Project (1KGP) (Consortium et al.,2012) and the African Genome Variation Project (AGVP) (Gurdasani et al.,2015) and used for analysis.

| **Population**  **label** | **Ethnic group** | **Population description** | **Total Samples** |
| --- | --- | --- | --- |
| **AFR** | Afro-Asiatic_Semitic | Amhara:Ethiopia | 22 |
|  | African-American | Americans of African Ancestry in SW USA (ASW) | 60 |
|  | African-Caribbean | African Caribbeans in Barbado (ACB) | 96 |
|  | Afro-Asiatic | Al-Gharbiyah, NA, Monufia, Kafrel-Sheikh, Mansoura, Alexandria, Dakahlia, Samanoud, Al-Buhayrah, Minya, AlSharqia, El-Mahalla all from Egypt | 99 |
|  | Afro-Asiatic_Cushitic | Oromo, Somali from Ethiopia | 47 |
|  | Afro-Asiatic_Omotic | Wolayta from Ethiopia | 24 |
|  | Khoe-San | Khoe-San:Khoesan | 84 |
|  | Niger_Congo_Bantu | Baganda, Banyarwanda, Barundi, RwandeseU- gandan, Banyankole:Uganda Bakiga, Mutan- zania, Basoga, other uganda gwas unknown, Mutooro, Batooro, Nyanjiro (Tanzania) from Uganda and Luhya in Webuye, Kenya (LWK) | 2158 |
|  | Niger-Congo_Bantu_South | Zulu | 98 |
|  | Niger_Congo_Volta_Niger | Esan in Nigeria (ESN), Yoruba in Ibadan, Nigeria (YRI) | 205 |
|  | Niger_Congo_West | Gambian in Western Divisions in the Gambia (GWD), Mende in Sierra Leone (MSL) | 198 |
| **AMR** | Latin_American | Puerto Ricans from Puerto Rico (PUR), Colombians from Medellin, Colombia (CLM), Peruvians from Lima, Peru (PEL), Mexican Ancestry from Los Angeles USA (MXL) | 347 |
| **EUR** | European_center | British in England and Scotland (GBR) | 91 |
|  | European_North | Finnish in Finland (FIN) | 99 |
|  | European_South | Iberian Population in Spain (IBS), Toscani in Italia (TSI) | 214 |
|  | European_USA | Utah Residents with Northern and Western European Ancestry (CEU) | 99 |
| **EAS** | East_Asian | Southern Han Chinese (CHS), Chinese Dai in Xishuangbanna, China (CDX), Kinh in Ho Chi Minh City, Vietnam (KHV), Han Chinese in Beijing, China (CHB), Japanese in Tokyo, Japan (JPT) | 504 |
|  | South_Asian | Punjabi from Lahore, Pakistan (PJL), Bengali from Bangladesh (BEB) | 180 |
| **SAS** | UK_Indian | Sri Lankan Tamil from the UK (STU), Indian Telugu from the UK (ITU) | 204 |
|  | USA_Indian | Gujarati Indian from Houston, Texas (GIH) | 103 |
| **Total** | | | 4,932 |
