# Supplementary material for: Insilico Functional Analysis of Genome-Wide Dataset From 17,000 Individuals Identifies Candidate Malaria Resistance Genes Enriched in Malaria Pathogenic Pathways
Source: Front Genet. 2021 Nov 18;12:676960. doi: 10.3389/fgene.2021.676960 (PMC8639191; doi:10.3389/fgene.2021.676960)
Supplement: Supplementary file 15 [file Table10.DOCX]

| **Ensg** | **entrezID** | **HUGO** | **symbol** | **chromosome** | **Cytoband** | **start** | **end** | **Biotype** |
| --- | --- | --- | --- | --- | --- | --- | --- | --- |
| **ENSG00000058668** | 493 | *ATP2B4* | *ATP2B4* | 1 | q32.1 | 203595689 | 203713209 | Protein coding |
| **ENSG00000148296** | 6838 | *SURF6* | *SURF6* | 9 | q34.2 | 136197552 | 136203235 | Protein coding |
| **ENSG00000148297** | 6837 | *MED22* | *MED22* | 9 | q34.2 | 136205160 | 136214986 | Protein coding |
| **ENSG00000148290** | 6834 | *SURF1* | *SURF1* | 9 | q34.2 | 136218610 | 136223552 | Protein coding |
| **ENSG00000148291** | 6835 | *SURF2* | *SURF2* | 9 | q34.2 | 136223428 | 136228045 | Protein coding |
| **ENSG00000148248** | 6836 | *SURF4* | *SURF4* | 9 | q34.2 | 136228325 | 136242970 | Protein coding |
| **ENSG00000132109** | 6737 | *TRIM21* | *TRIM21* | 11 | p15.4 | 4406127 | 4414926 | Protein coding |
| **ENSG00000176925** | 119694 | *OR51F2* | *OR51F2* | 11 | p15.4 | 4842551 | 4843686 | Protein coding |
| **ENSG00000244734** | 3043 | *HBB* | *HBB* | 11 | p15.4 | 5246694 | 5250625 | Protein coding |
| **ENSG00000223609** | 3045 | *HBD* | *HBD* | 11 | p15.4 | 5253908 | 5256600 | Protein coding |
| **ENSG00000196565** | 3048 | *HBG2* | *HBG2* | 11 | p15.4 | 5274420 | 5667019 | Protein coding |
| **ENSG00000213931** | 3046 | *HBE1* | *HBE1* | 11 | p15.4 | 5289582 | 5526847 | Protein coding |
| **ENSG00000184881** | 79345 | *OR51B2* | *OR51B2* | 11 | p15.4 | 5344541 | 5345582 | Protein coding |

**S Table 2.** Candidate malaria resistance genes identified by both functional mapping (FUMA) and gene-based GWAS (PASCAL) analyses
